# Supplementary material for: From Guidelines to Practice: Case-Based Teaching Module to Improve MASLD Screening in Diabetes Care for Internal Medicine Residents
Source: MedEdPORTAL. 2026 Mar 24;22:11586. doi: 10.15766/mep_2374-8265.11586 (PMC13008821; doi:10.15766/mep_2374-8265.11586)
Supplement: Supplementary file 1 — MASLD Screening Education Module.pptxMASLD Survey.docx [file mep_2374-8265.11586-s001.zip › B. MASLD Survey.docx]

**MASLD Survey**

Adapted from: Younossi, Z. M. et al. A Global Survey of Physicians Knowledge About Nonalcoholic Fatty Liver Disease. Clinical Gastroenterology and Hepatology 20, e1456–e1468 (2022).

**Enter your unique identifier:**

First 2 letters of mother’s maiden name in all caps + 2-digit representation of the month you were born (eg. June → 06) + number of siblings you have (including half siblings, living or deceased; if none, write X).

Example: enter DO123 for maiden name Doe + birth month December + 3 siblings

Please make sure to take note of this as the same identifier will be used to link your pre- and post-surveys.

**PGY class:**

1. PGY-1
2. PGY-2
3. PGY-3

Metabolic dysfunction associated steatotic liver disease (MASLD), formerly known as nonalcoholic fatty liver disease (NAFLD), is the fastest growing cause of chronic liver disease worldwide. As the obesity and diabetes epidemic continues and the general populations in most countries become older, MASLD and its progressive subtype metabolic dysfunction associated steatohepatitis (MASH), formerly known as nonalcoholic steatohepatitis (NASH), will have a significant impact. We want to assess the awareness among internal medicine residents about MASLD and MASH in the primary care setting.

1. In your opinion, is MASLD an important public health problem?
   1. Yes
   2. No
2. What is the prevalence of MASLD in the general population worldwide?
   1. < 10%
   2. 10-20%
   3. 21-30%
   4. >30%
   5. I don’t know
3. What is the prevalence of MASLD in the general population in the US?
   1. < 10%
   2. 10-20%
   3. 21-30%
   4. >30%
   5. I don’t know
4. What is the prevalence of MASLD in patients with diabetes?
   1. <10%
   2. 11-30%
   3. 31-60%
   4. >60%
   5. I don’t know
5. What is the prevalence of MASH in patients with diabetes?
   1. < 10%
   2. 10-20%
   3. 21-30%
   4. >30%
   5. I don’t know
6. Which type of individual do you screen for MASLD? (check all that apply)
   1. Everyone
   2. Diabetes
   3. Hypertension
   4. Dyslipidemia
   5. Sleep apnea
   6. Hypothyroidism
   7. Cardio-vascular disease
   8. Polycystic ovary syndrome
   9. Unexplained raised ALT
   10. Cryptogenic liver disease
   11. I do not screen for MASLD
7. In your practice, do you recommend screening patients with diabetes for MASLD?
   1. Yes
   2. No
8. In your opinion, what is the initial assessment of patients with suspected MASLD? (Please check all that apply)
   1. Elevated liver enzymes
   2. Exclusion of other liver diseases
   3. Controlled Attenuation Parameter
   4. Liver biopsy
   5. Liver imaging such as ultrasound, MRI or CT scan
   6. None
9. What is the role of fibroscan in MASLD? (Please check all that apply)
   1. Estimating liver stiffness and stage of fibrosis
   2. Estimating amount fat in the liver
   3. Best prognostic test
   4. No value
10. Which of the following is considered the gold standard for the diagnosis of MASH?
    1. Hepatic ultrasound
    2. Controlled Attenuation Parameter
    3. Magnetic resonance
    4. Liver biopsy
    5. None
11. Do you refer cases of MASLD to a gastroenterologist or hepatologist?
    1. Yes
    2. No
12. When do you send a patient with MASLD to a specialist? (Please check all that apply)
    1. At risk for steatohepatitis or cirrhosis
    2. Other conditions that cause steatohepatitis that can’t be excluded
    3. Other conditions that cause steatohepatitis coexist
    4. Presence of metabolic syndrome
    5. High FIB-4 score (≥ 1.3)

**SURVEY SELECTION**

1. I am taking this survey and…
   1. I am about to attend the MASLD teaching module [if selected, skip to end of survey]
   2. I have just completed the MASLD teaching module [if selected, show question 14 only]
   3. I completed the MASLD teaching module weeks ago [if selected, show questions 14-16]

**POST-SURVEY ASSESSMENT**

1. The MASLD module taught me new clinical knowledge
   1. Agree
   2. Disagree
2. I have changed my MASLD screening practices as a result of this teaching module
   1. Agree
   2. Disagree
3. I have changed my referral practices for patients with suspected MASLD as a result of this teaching module
   1. Agree
   2. Disagree
