# Supplementary material for: From Guidelines to Practice: Case-Based Teaching Module to Improve MASLD Screening in Diabetes Care for Internal Medicine Residents
Source: MedEdPORTAL. 2026 Mar 24;22:11586. doi: 10.15766/mep_2374-8265.11586 (PMC13008821; doi:10.15766/mep_2374-8265.11586)
Supplement: Supplementary file 1 — MASLD Screening Education Module.pptxMASLD Survey.docx [file mep_2374-8265.11586-s001.zip › A. MASLD Screening Education Module.pptx]

## Slide 1
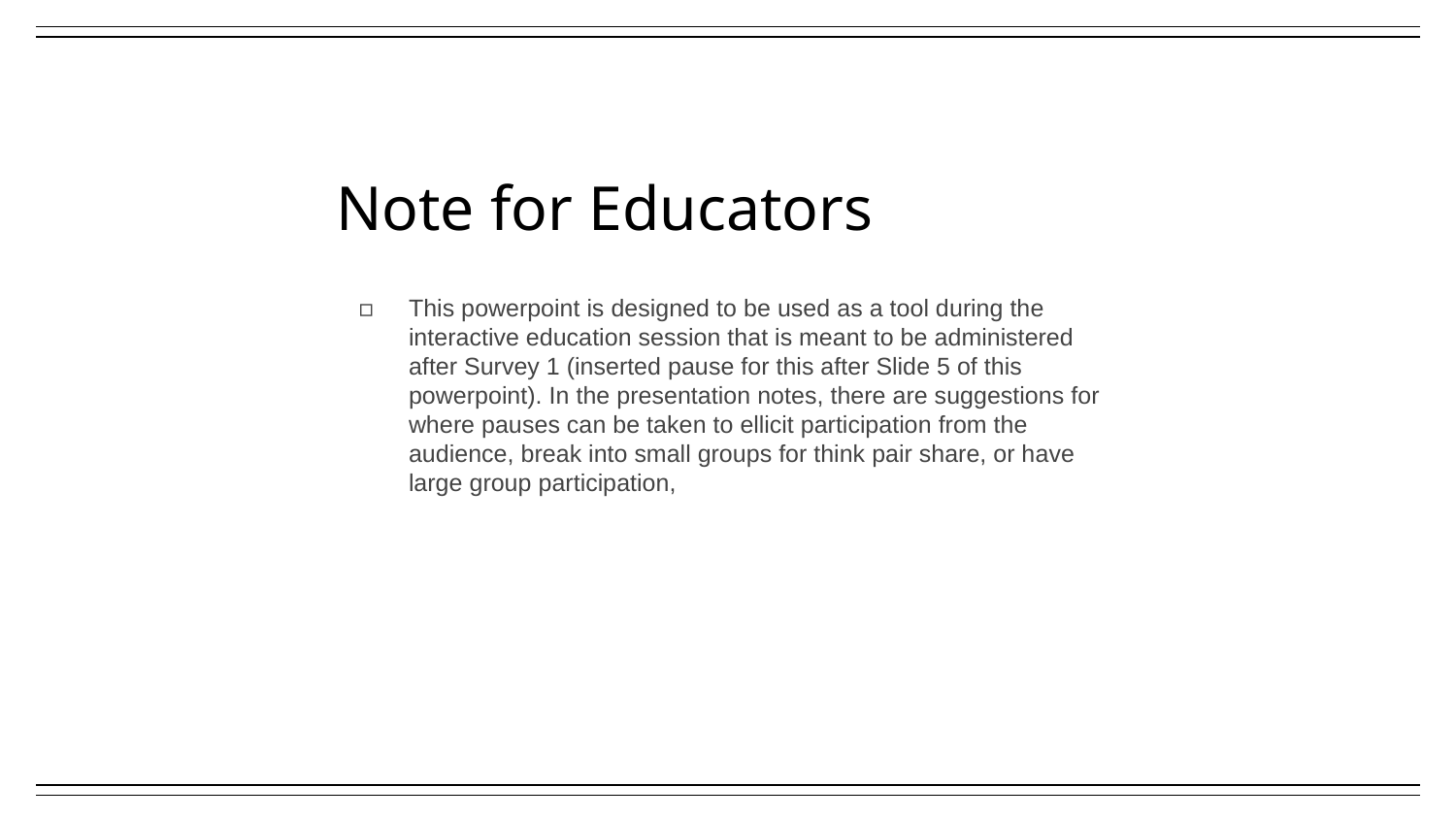

# Note for Educators
This powerpoint is designed to be used as a tool during the interactive education session that is meant to be administered after Survey 1 (inserted pause for this after Slide 5 of this powerpoint). In the presentation notes, there are suggestions for where pauses can be taken to ellicit participation from the audience, break into small groups for think pair share, or have large group participation,

## Slide 2
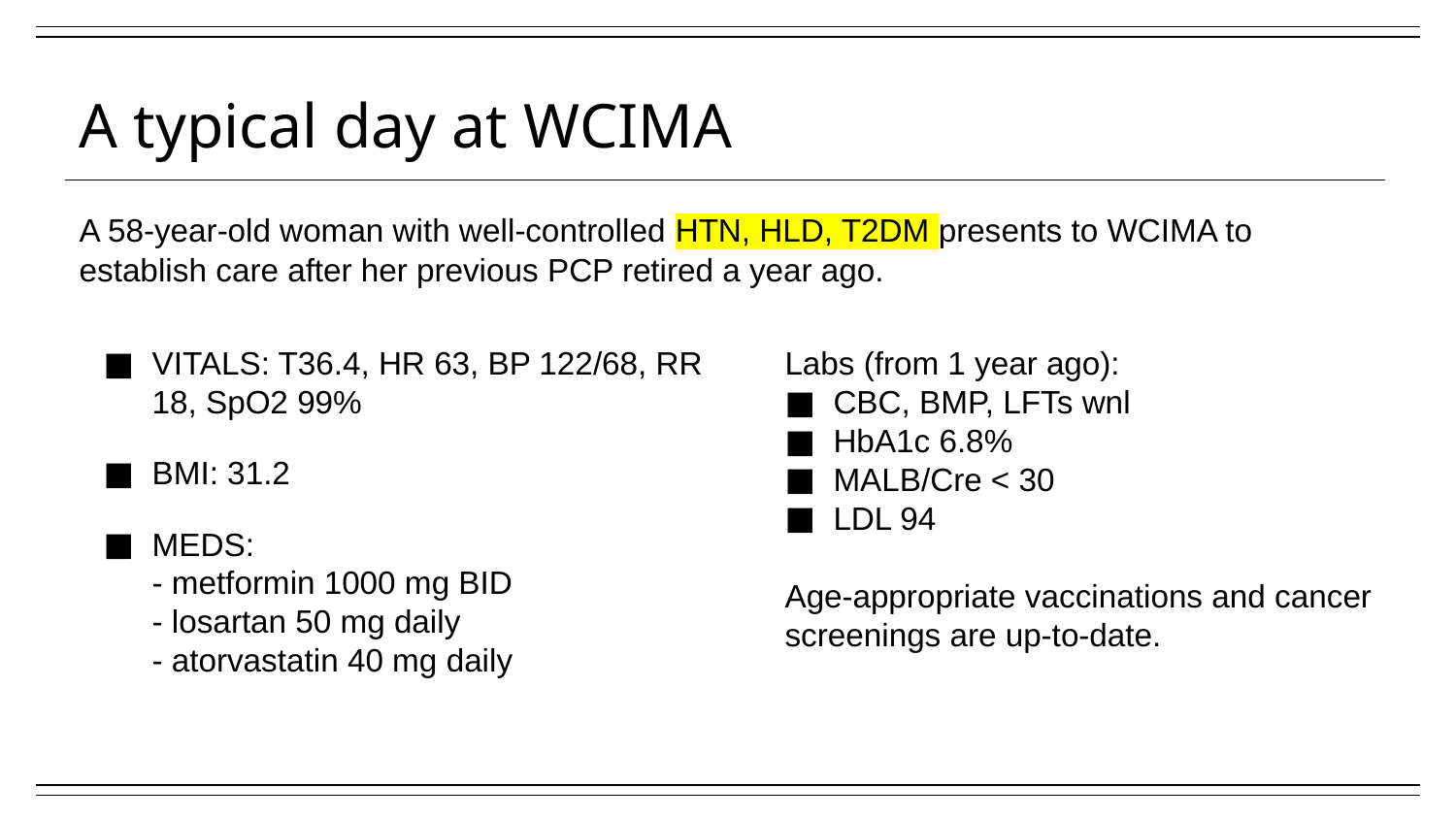

# A typical day at WCIMA
A 58-year-old woman with well-controlled HTN, HLD, T2DM presents to WCIMA to establish care after her previous PCP retired a year ago.
VITALS: T36.4, HR 63, BP 122/68, RR 18, SpO2 99%
BMI: 31.2
MEDS:
- metformin 1000 mg BID
- losartan 50 mg daily
- atorvastatin 40 mg daily
Labs (from 1 year ago):
CBC, BMP, LFTs wnl
HbA1c 6.8%
MALB/Cre < 30
LDL 94
Age-appropriate vaccinations and cancer screenings are up-to-date.

## Slide 3
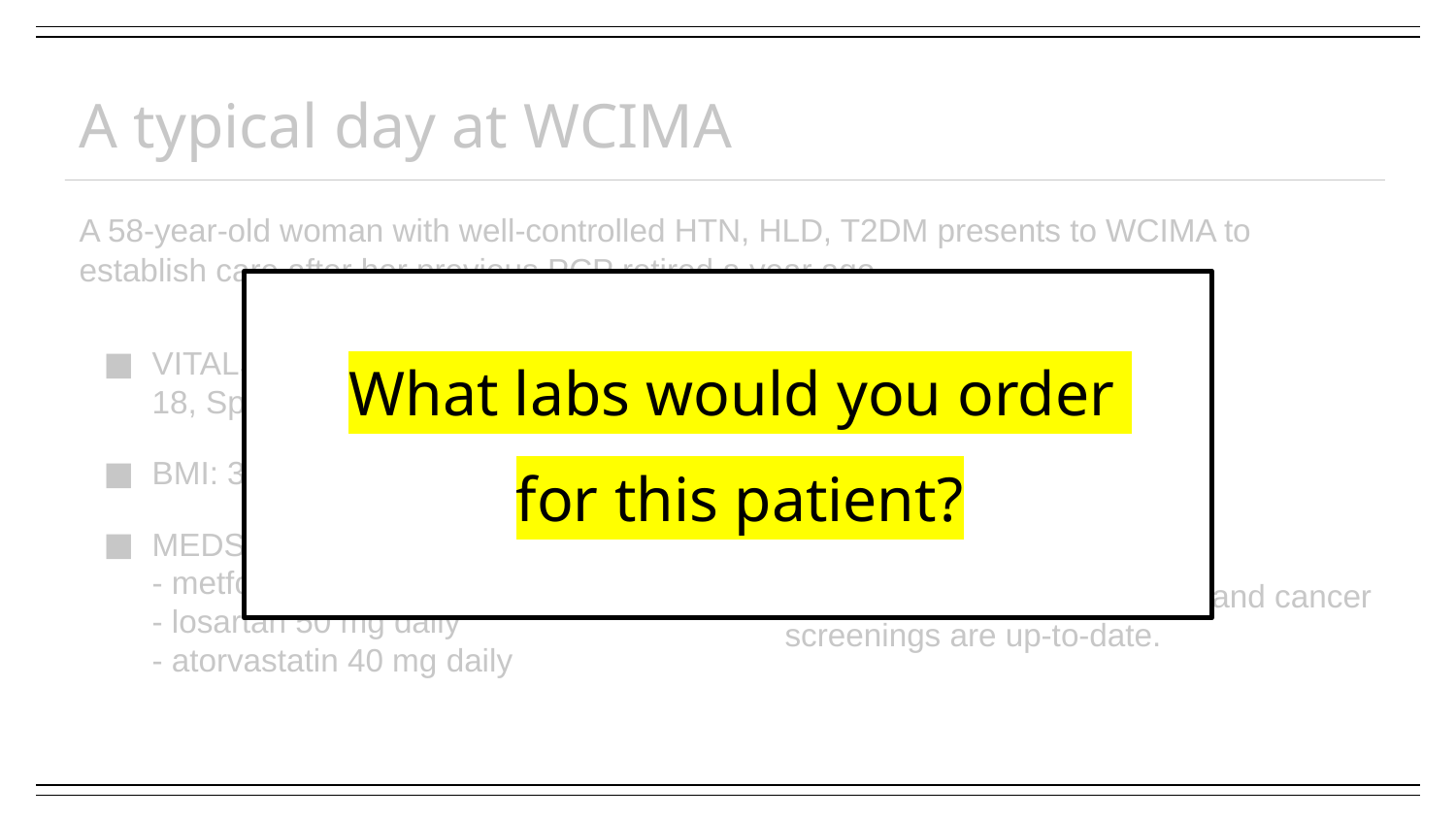

# A typical day at WCIMA
A 58-year-old woman with well-controlled HTN, HLD, T2DM presents to WCIMA to establish care after her previous PCP retired a year ago.
What labs would you order
for this patient?
VITALS: T36.4, HR 63, BP 122/68, RR 18, SpO2 99%
BMI: 31.2
MEDS:
- metformin 1000 mg BID
- losartan 50 mg daily
- atorvastatin 40 mg daily
Labs (from 1 year ago):
CBC, BMP, LFTs wnl
HbA1c 6.8%
MALB/Cre < 30
LDL 94
Age-appropriate vaccinations and cancer screenings are up-to-date.

## Slide 4
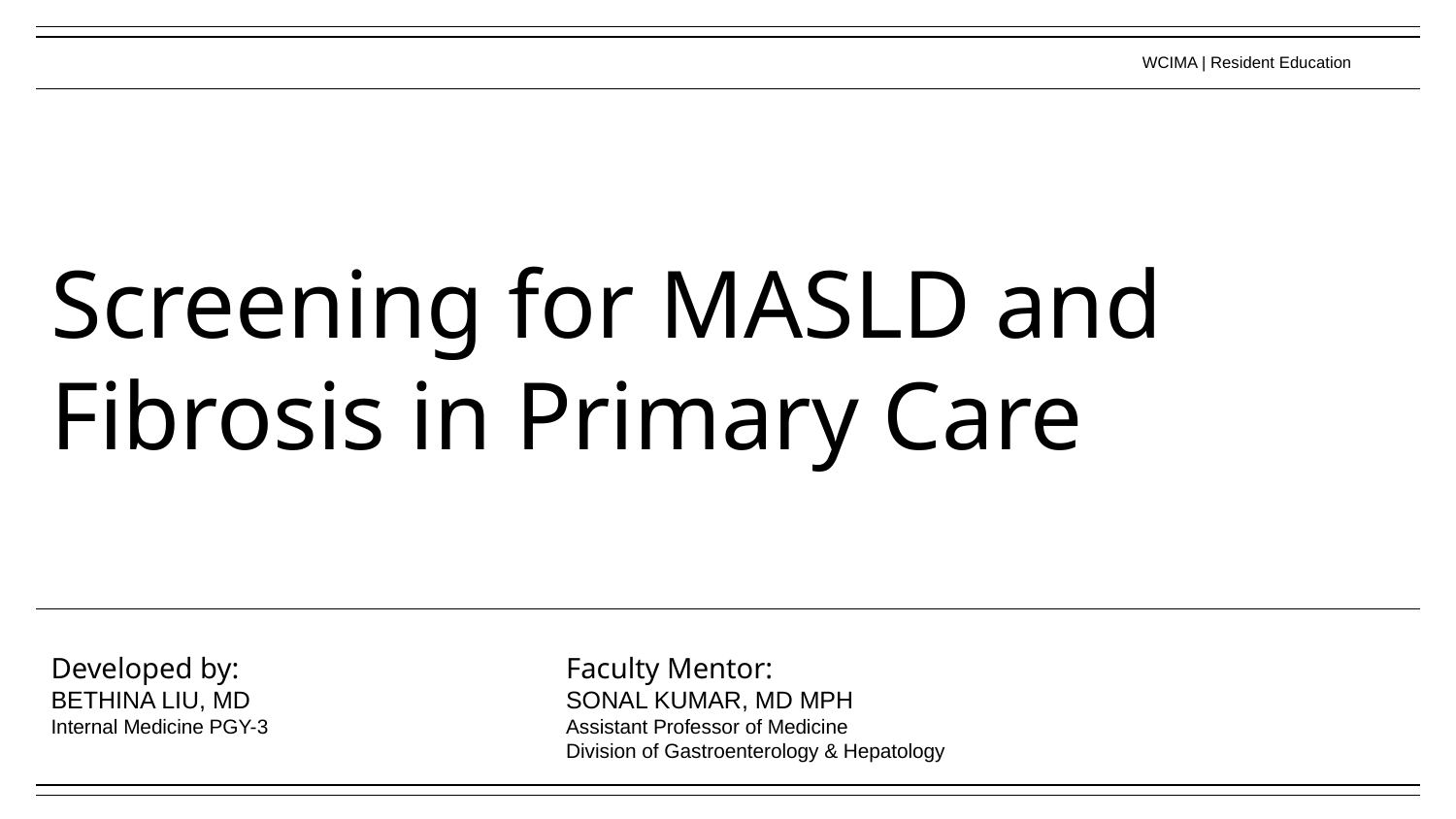

WCIMA | Resident Education
# Screening for MASLD and Fibrosis in Primary Care
Developed by:
BETHINA LIU, MD
Internal Medicine PGY-3
Faculty Mentor:
SONAL KUMAR, MD MPH
Assistant Professor of Medicine
Division of Gastroenterology & Hepatology

## Slide 5
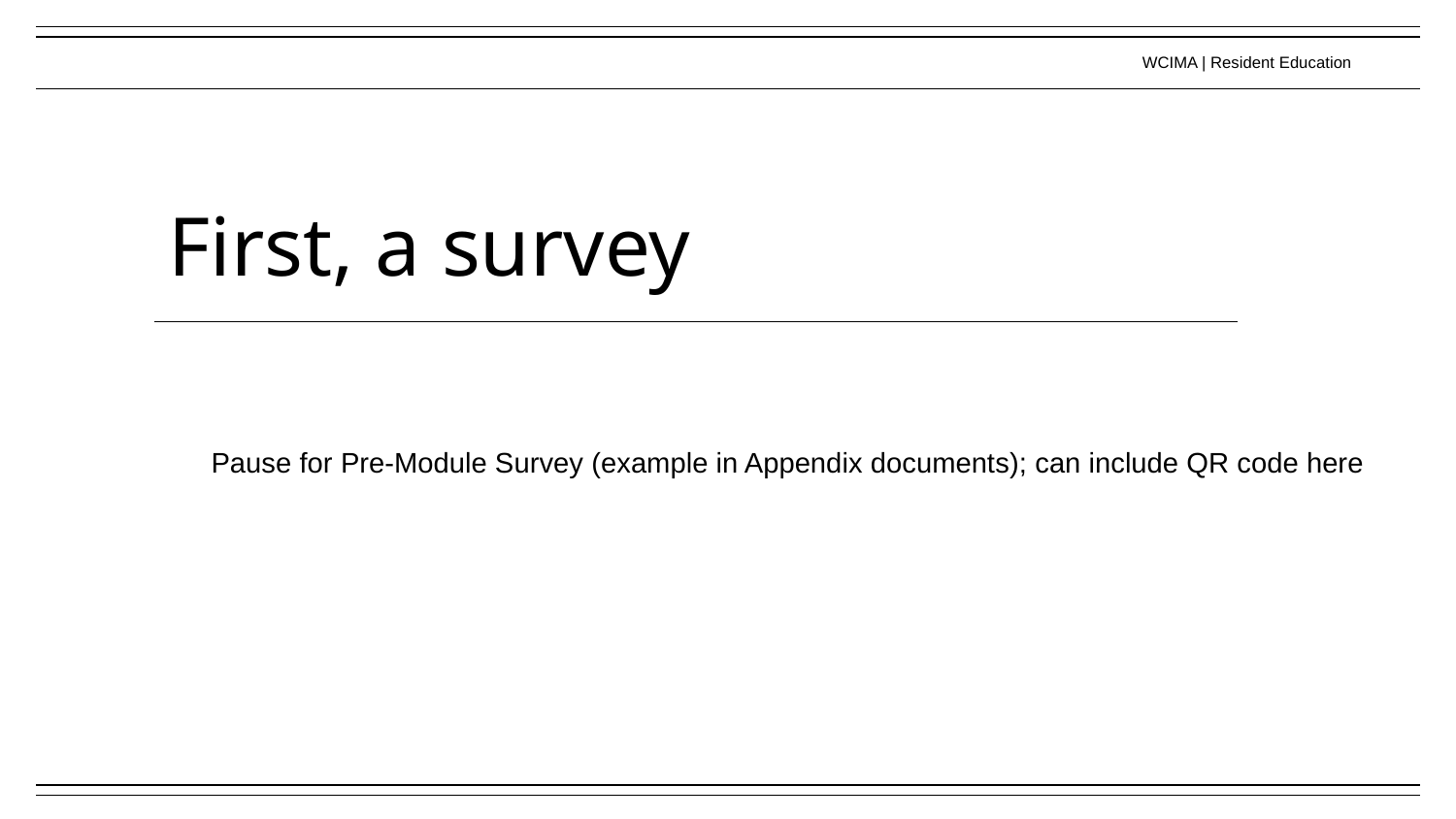

WCIMA | Resident Education
# First, a survey
Pause for Pre-Module Survey (example in Appendix documents); can include QR code here

## Slide 6
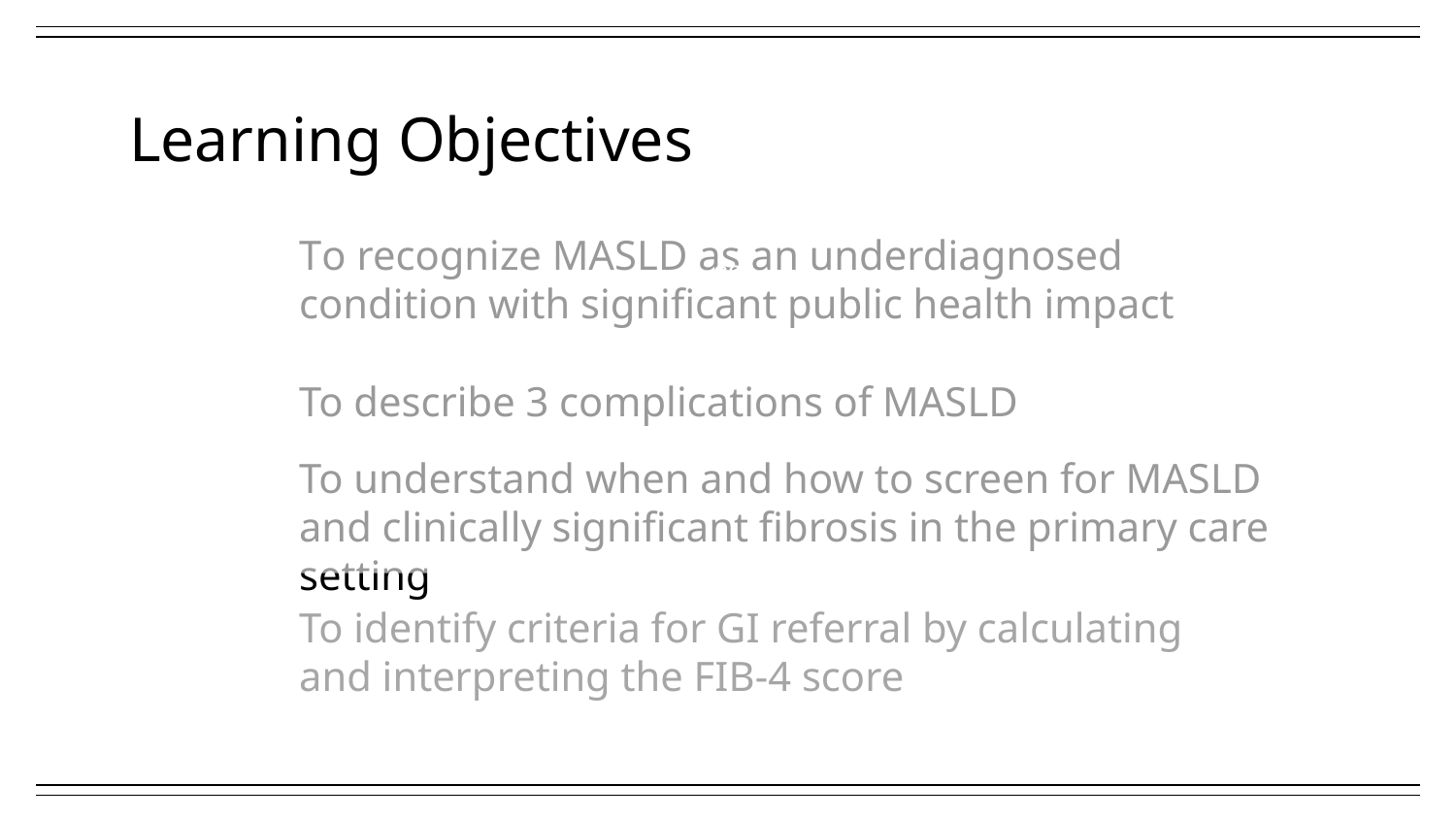

Learning Objectives
cc
To recognize MASLD as an underdiagnosed condition with significant public health impact
01
To describe 3 complications of MASLD
02
To understand when and how to screen for MASLD and clinically significant fibrosis in the primary care setting
03
To identify criteria for GI referral by calculating and interpreting the FIB-4 score
04

## Slide 7
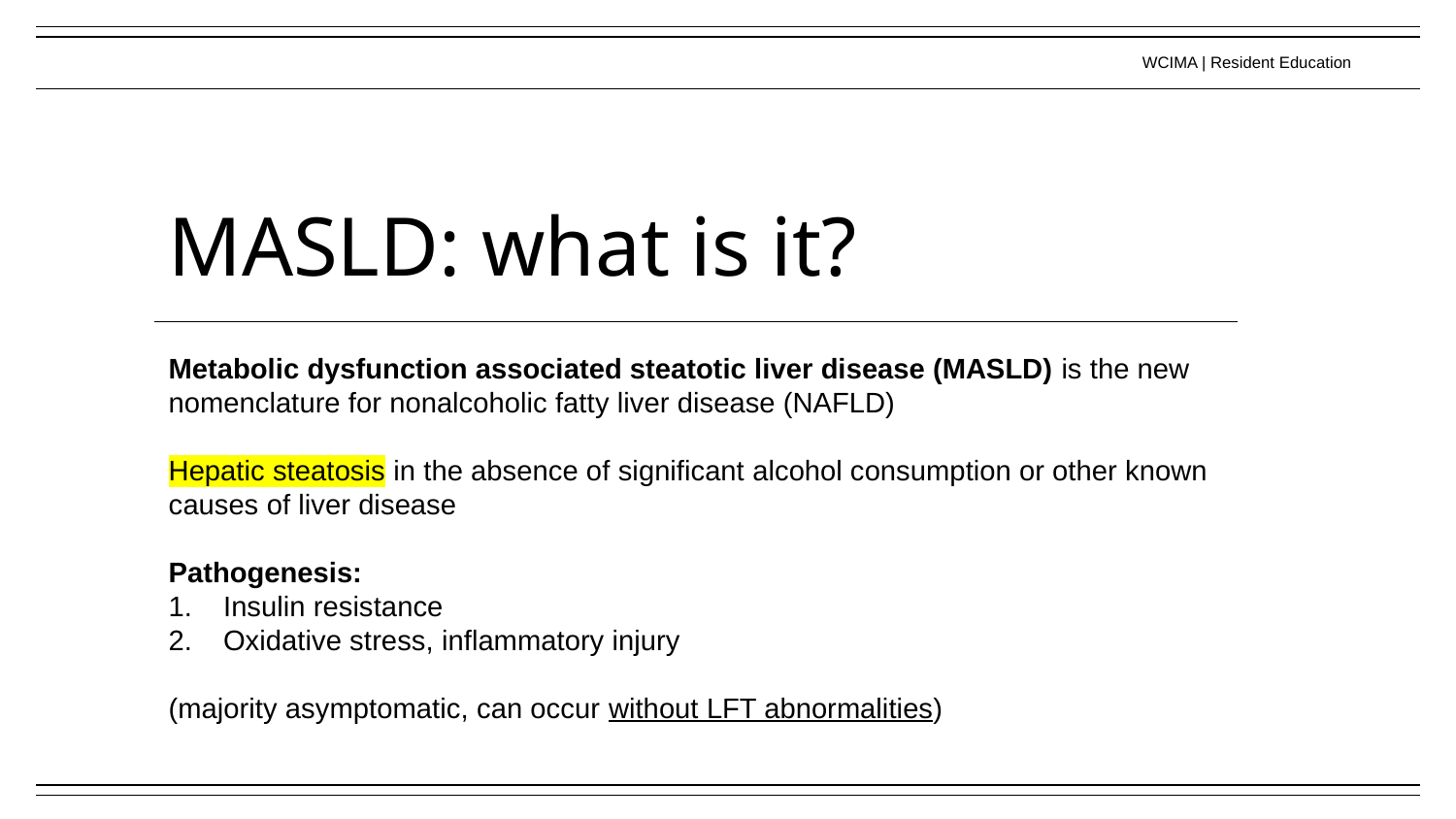

WCIMA | Resident Education
# MASLD: what is it?
Metabolic dysfunction associated steatotic liver disease (MASLD) is the new nomenclature for nonalcoholic fatty liver disease (NAFLD)
Hepatic steatosis in the absence of significant alcohol consumption or other known causes of liver disease
Pathogenesis:
Insulin resistance
Oxidative stress, inflammatory injury
(majority asymptomatic, can occur without LFT abnormalities)

## Slide 8
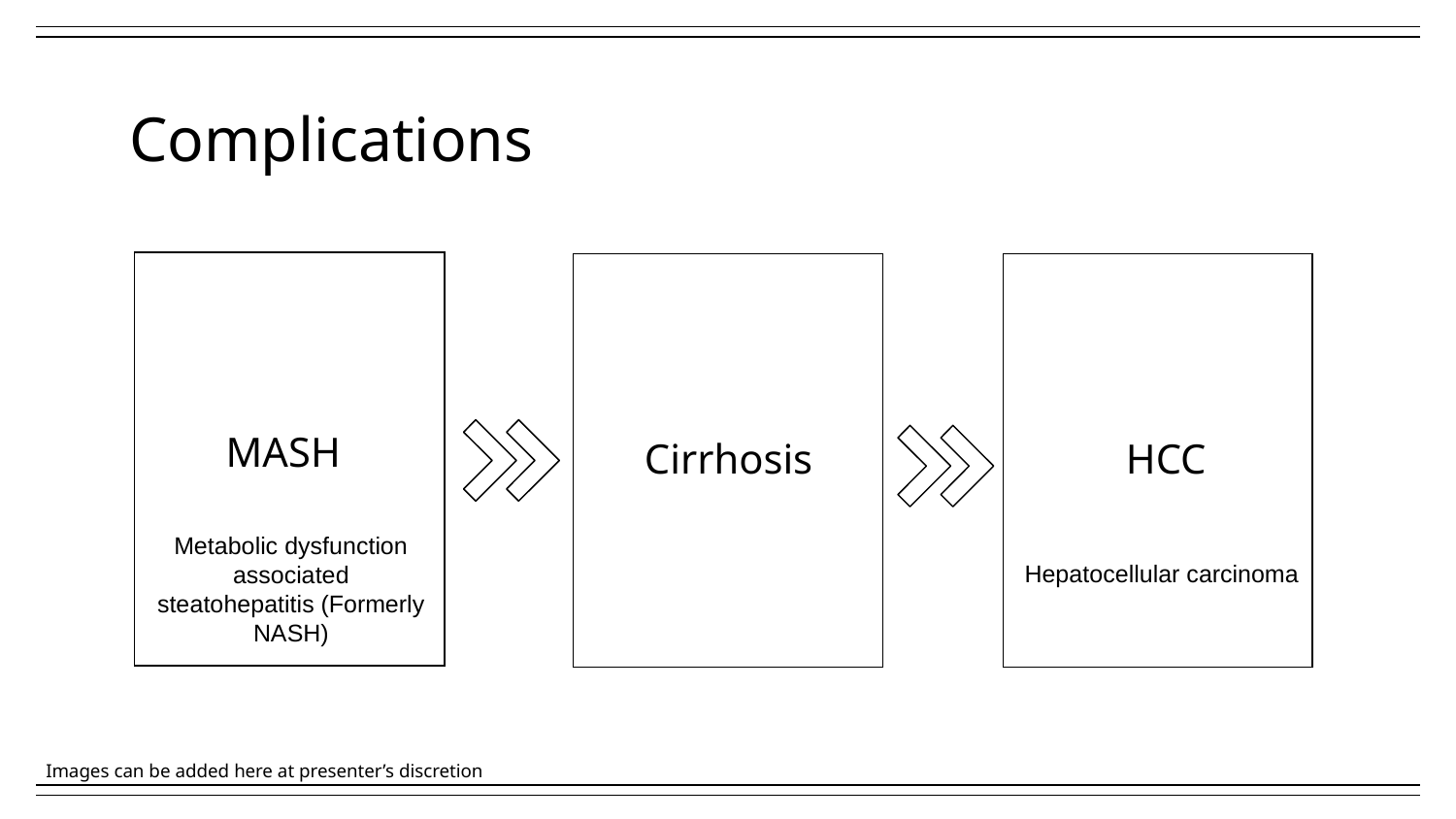

# Complications
MASH
Cirrhosis
HCC
Hepatocellular carcinoma
Metabolic dysfunction associated steatohepatitis (Formerly NASH)
Images can be added here at presenter’s discretion

## Slide 9
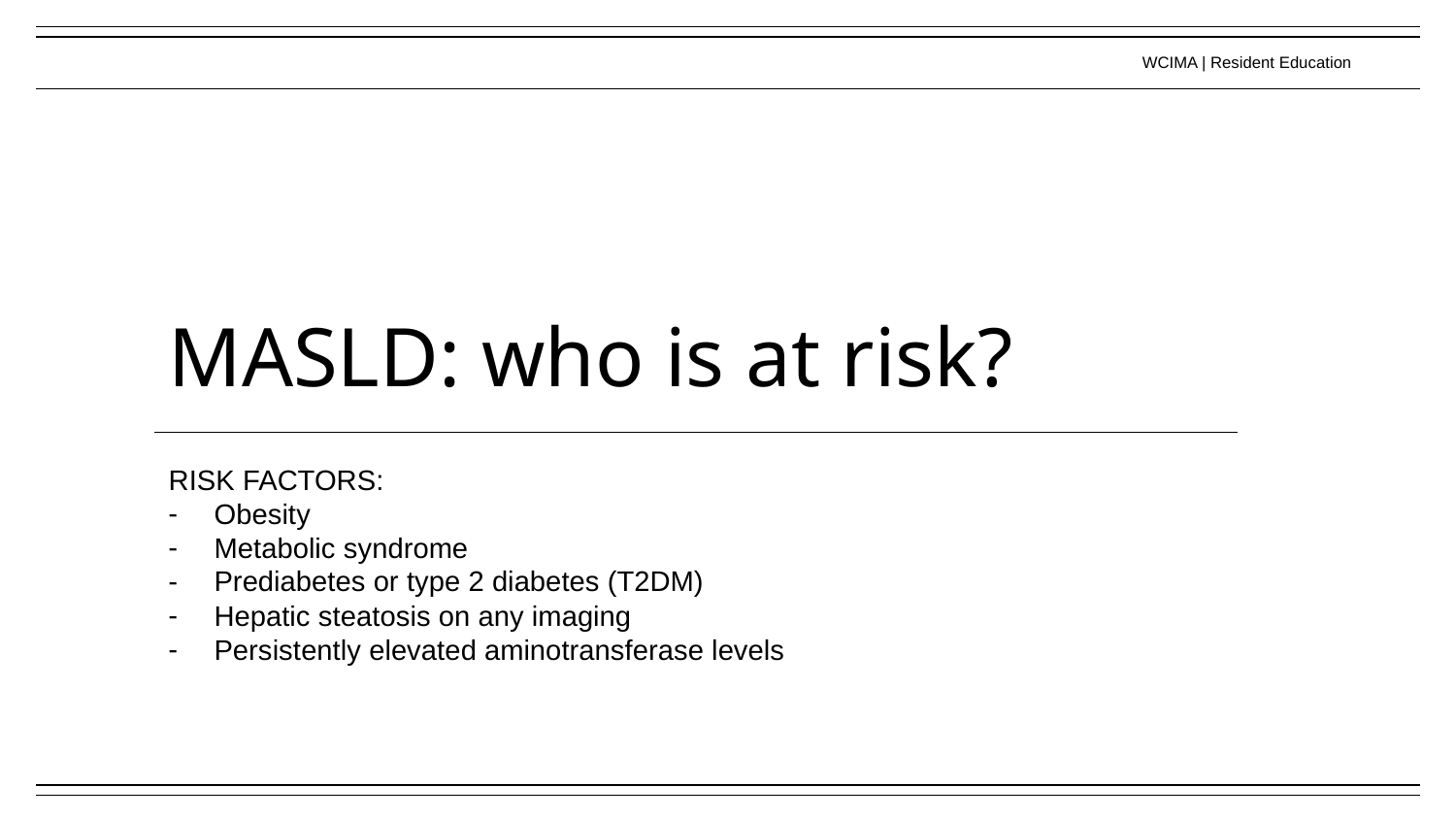

WCIMA | Resident Education
# MASLD: who is at risk?
RISK FACTORS:
Obesity
Metabolic syndrome
Prediabetes or type 2 diabetes (T2DM)
Hepatic steatosis on any imaging
Persistently elevated aminotransferase levels

## Slide 10
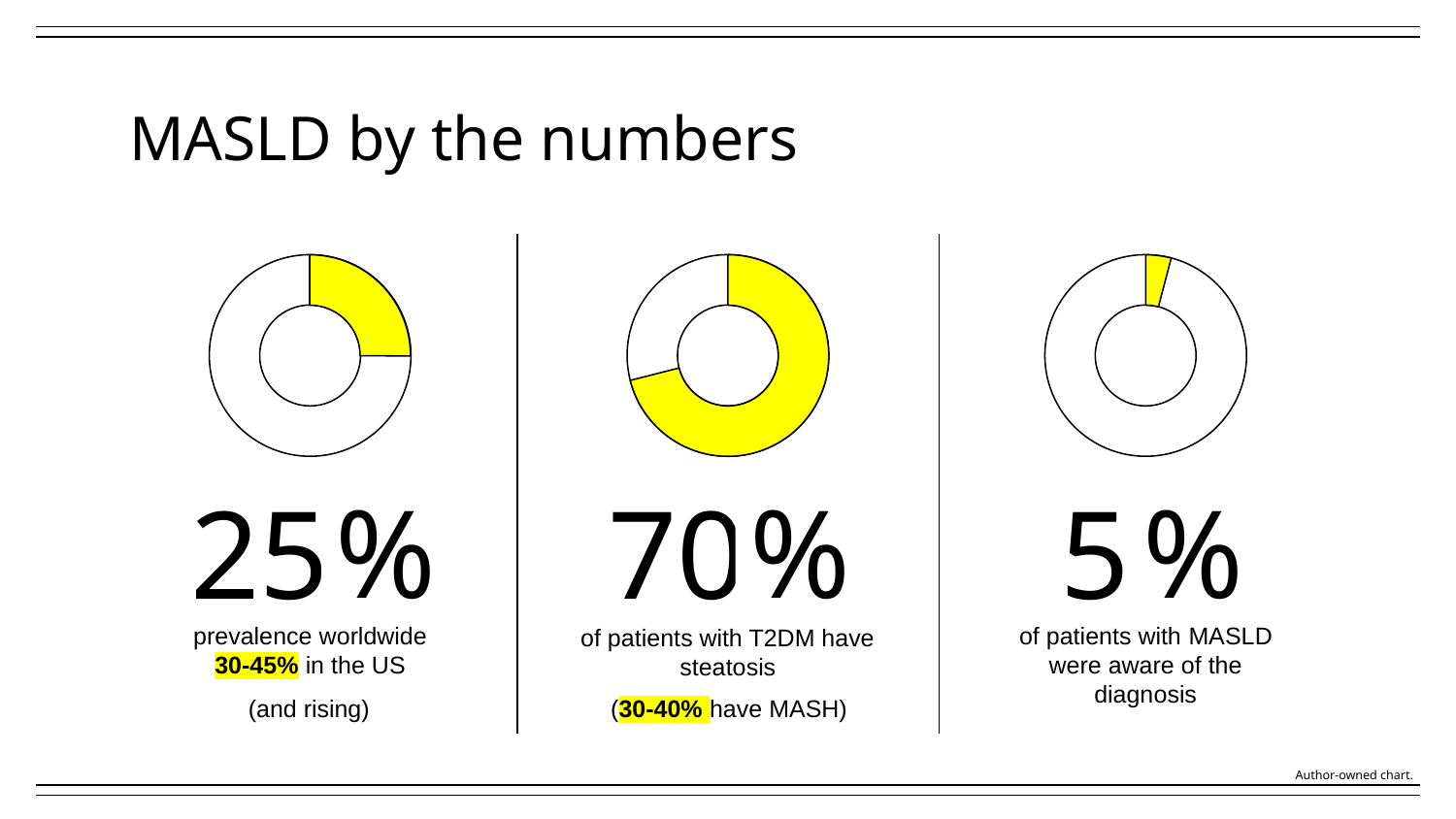

# MASLD by the numbers
%
%
%
25%
70%
5%
prevalence worldwide
30-45% in the US
of patients with MASLD were aware of the diagnosis
of patients with T2DM have steatosis
(and rising)
(30-40% have MASH)
Author-owned chart.

## Slide 11
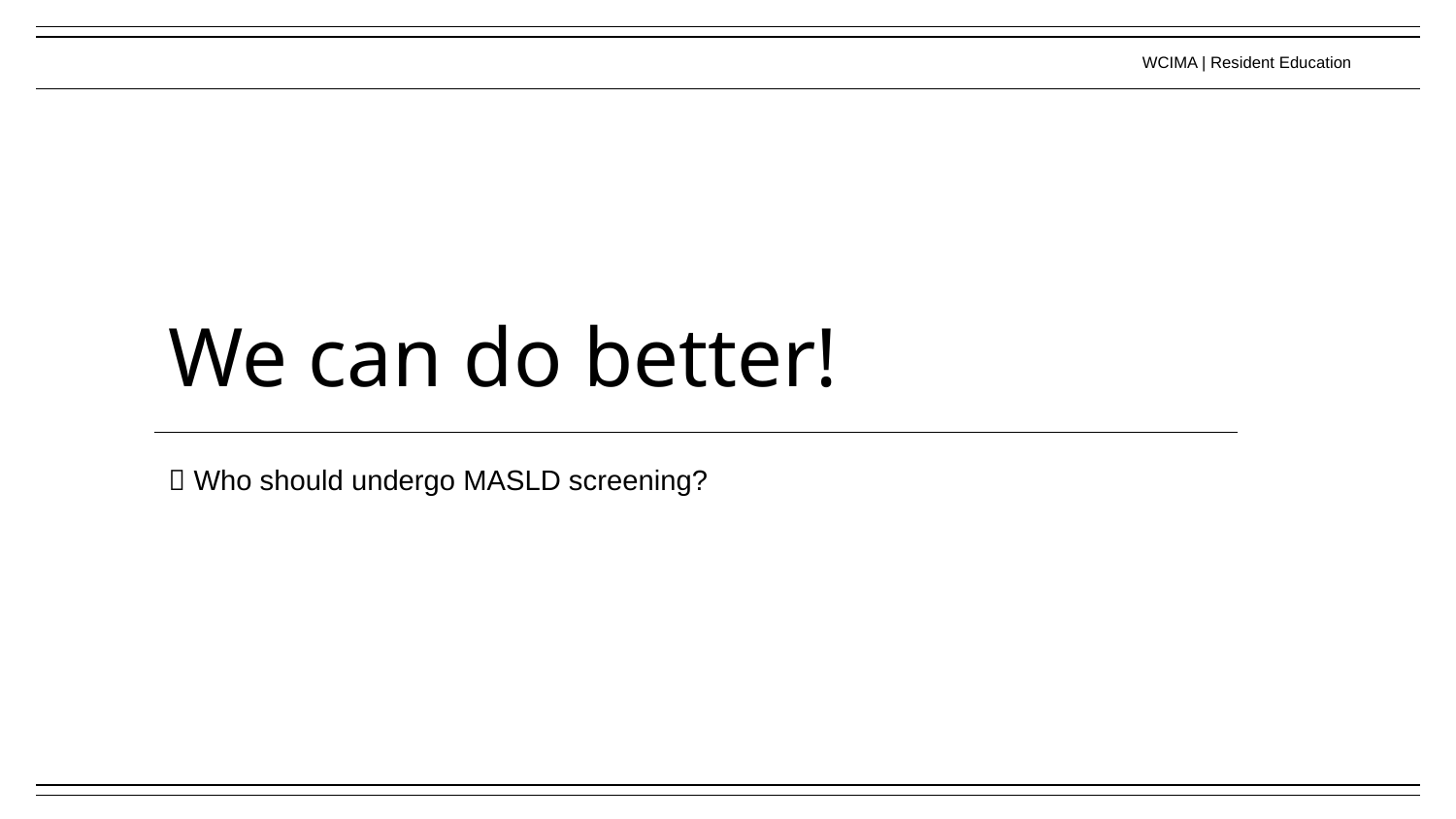

WCIMA | Resident Education
# We can do better!
 Who should undergo MASLD screening?

## Slide 12
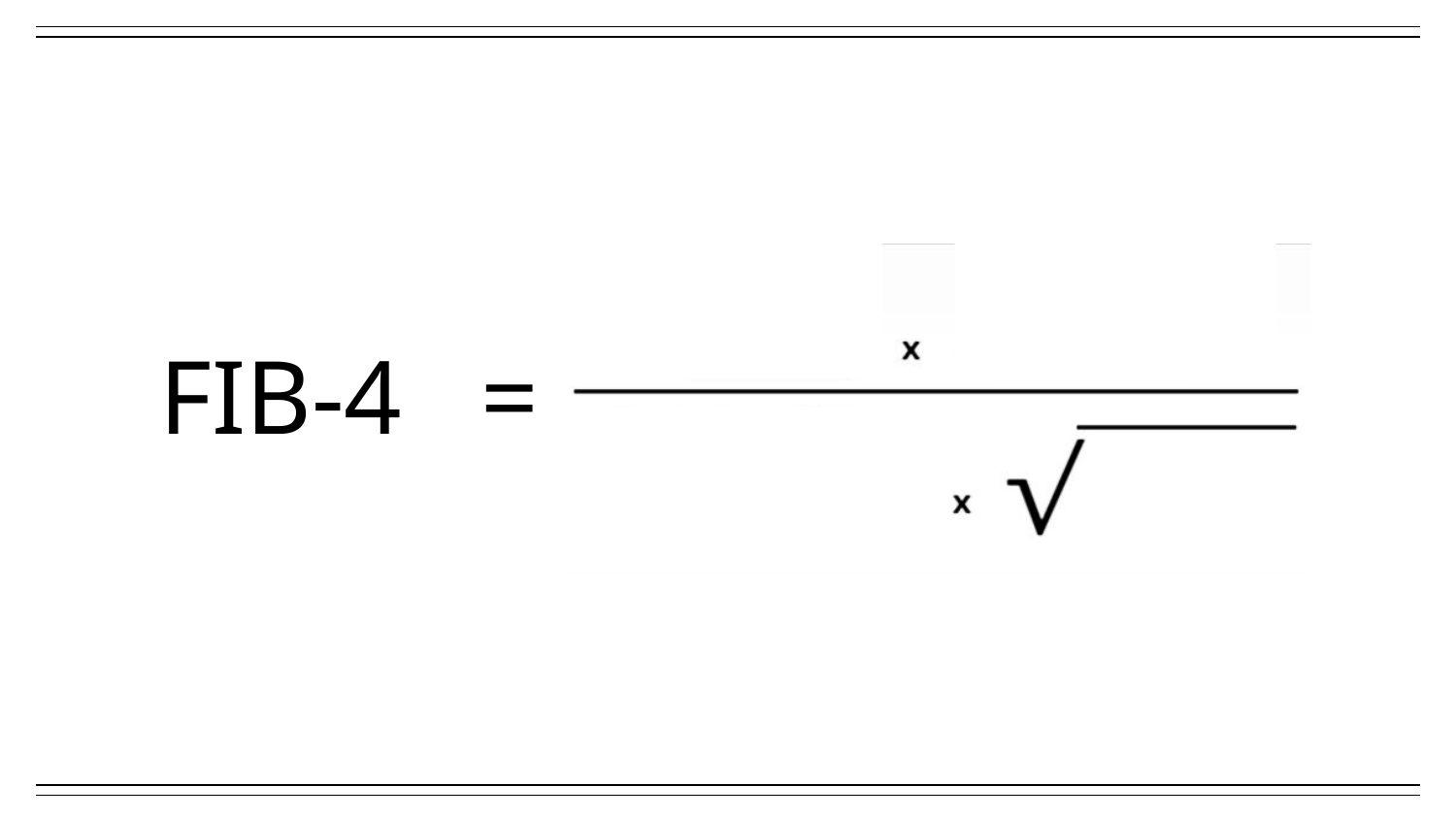

# FIB-4 =

## Slide 13
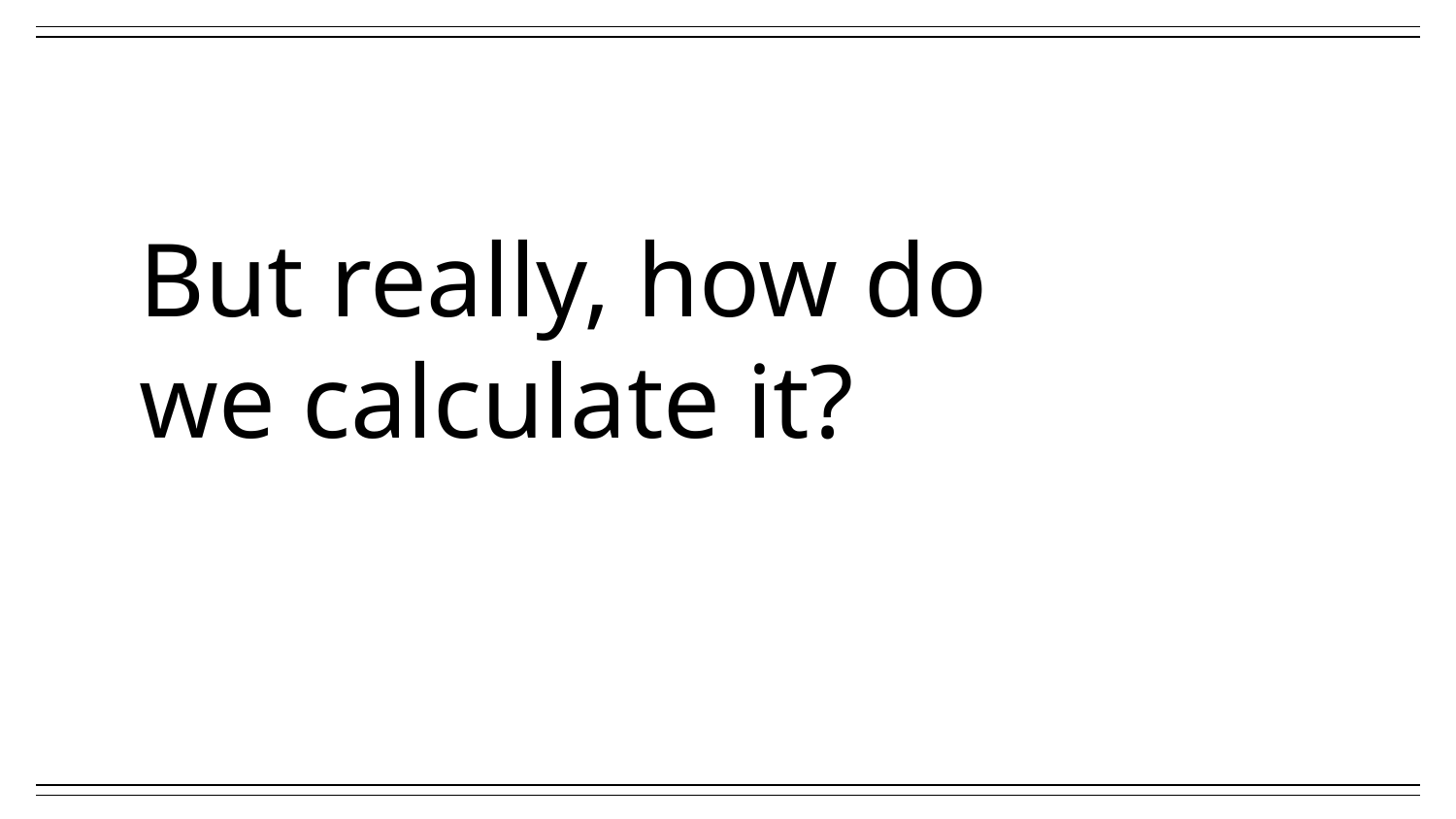

But really, how do we calculate it?
# FIB-4 =

## Slide 14
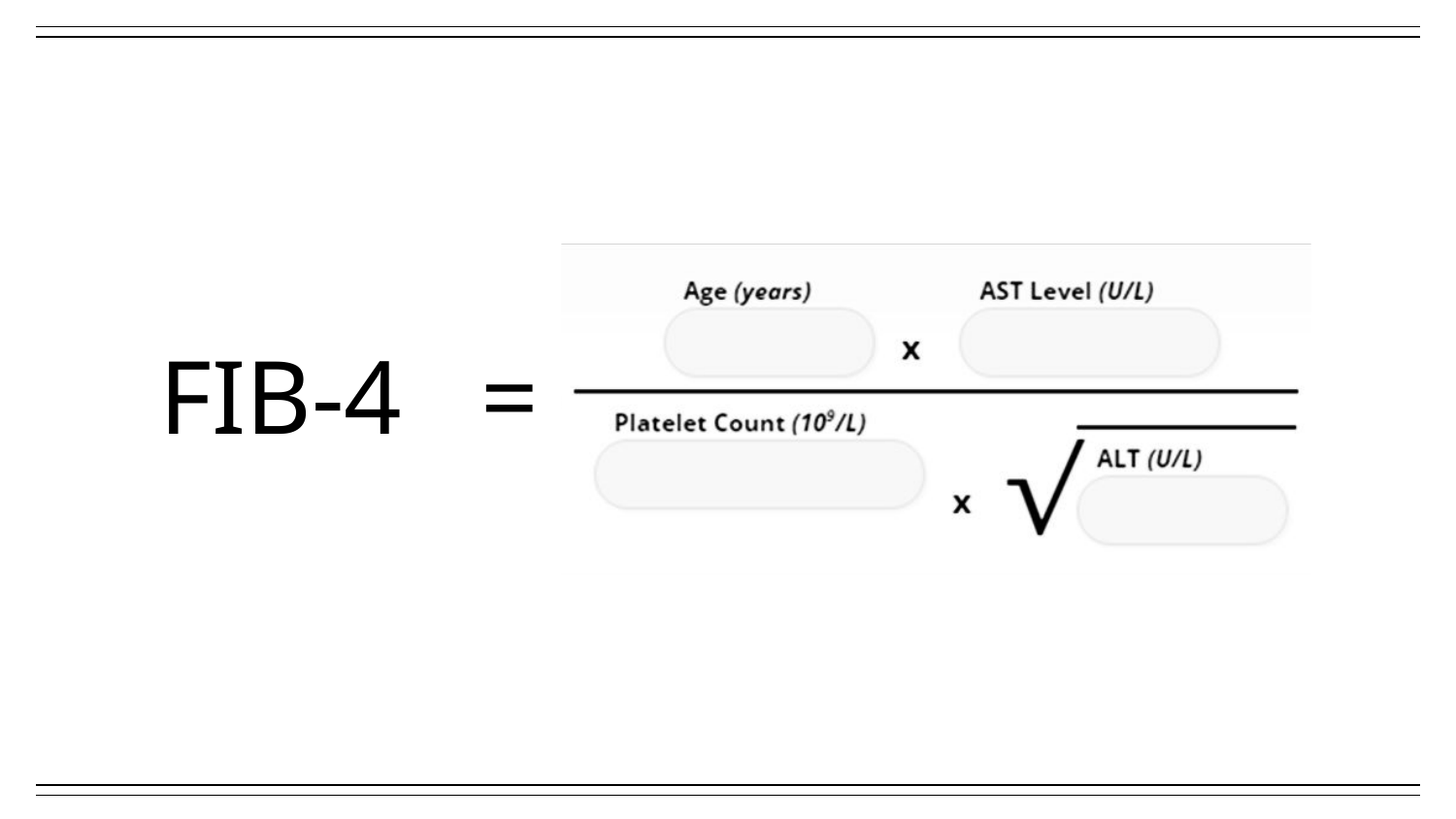

# FIB-4 =

## Slide 15
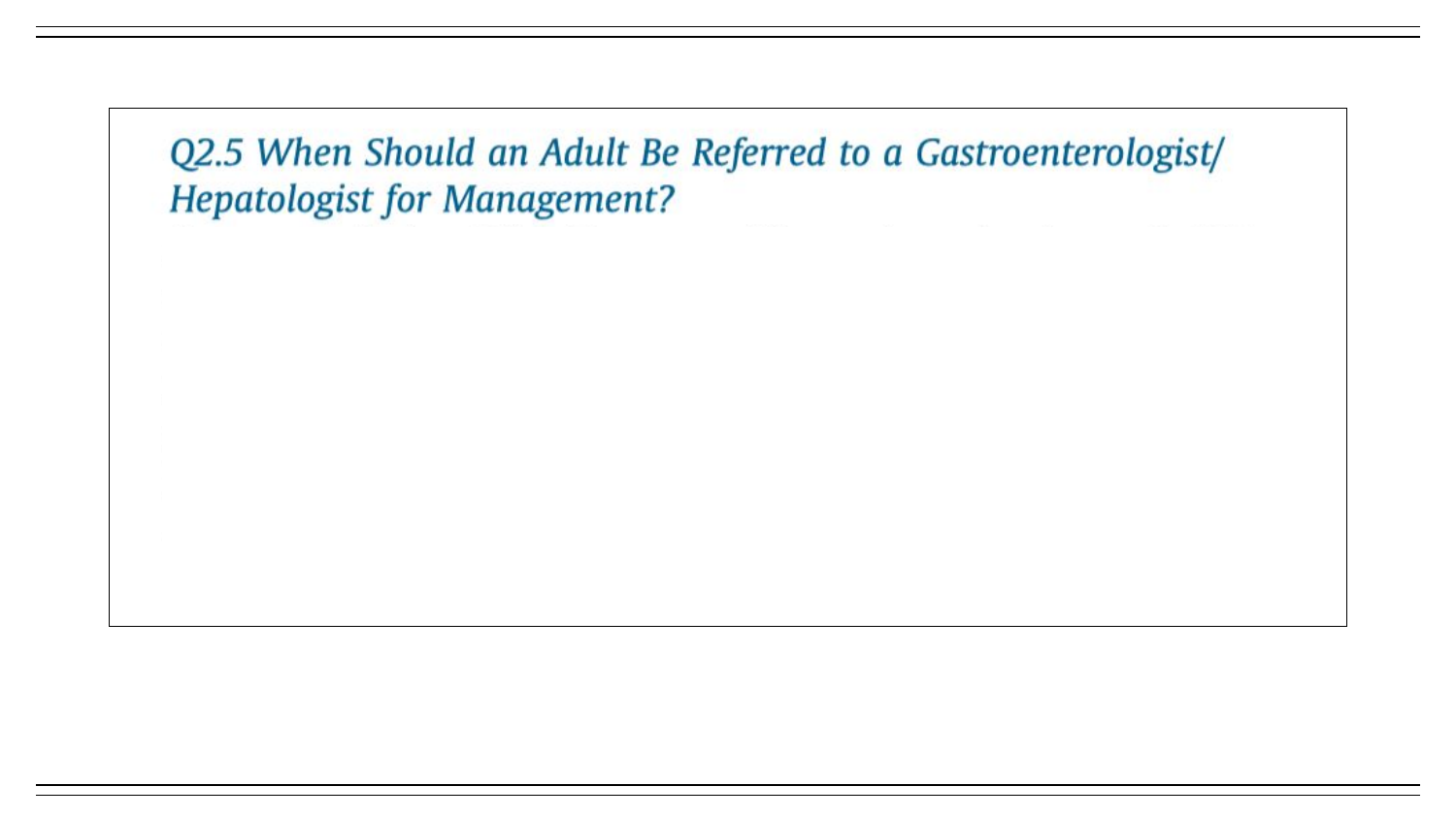

## Slide 16
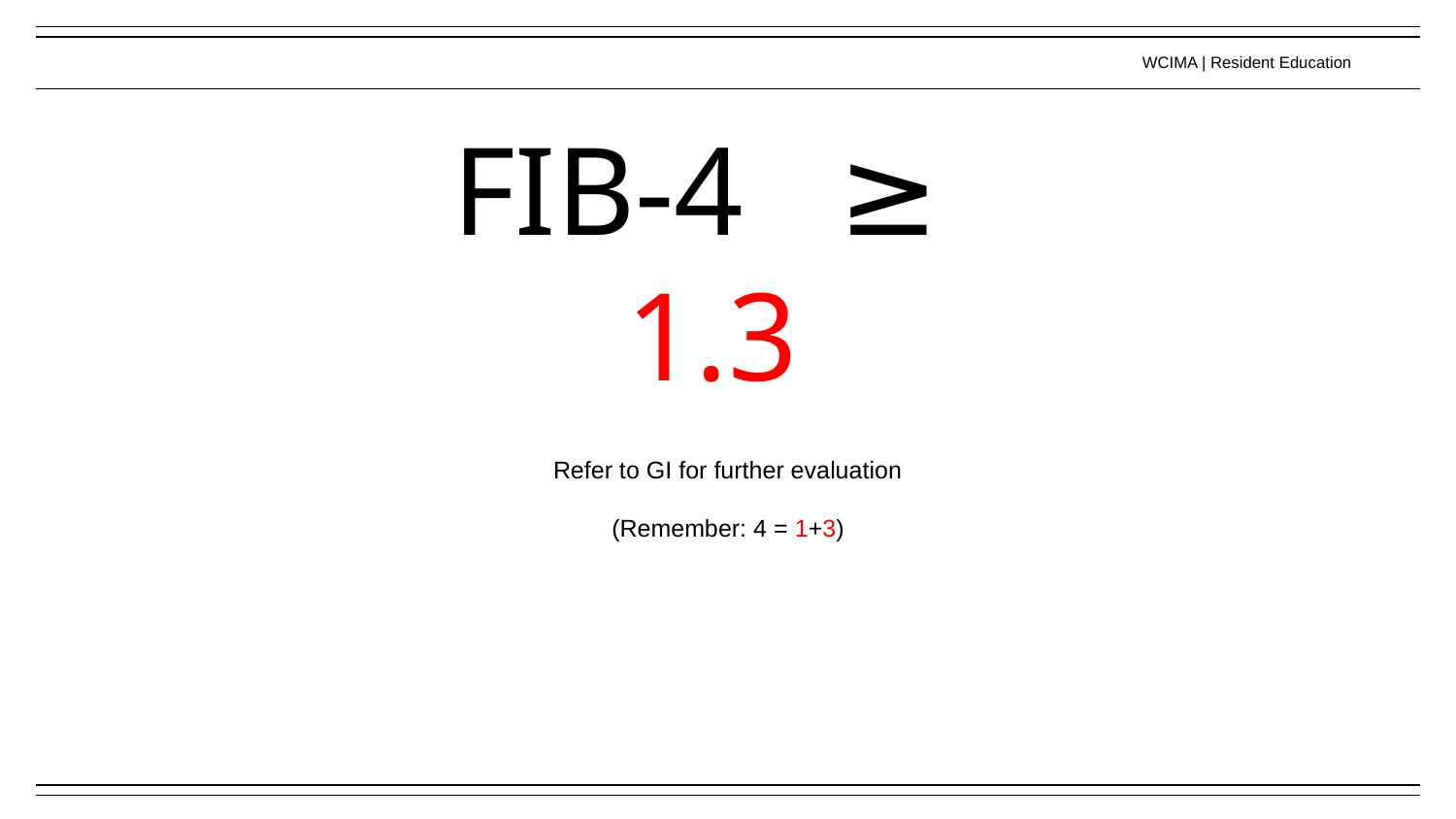

WCIMA | Resident Education
# FIB-4 ≥ 1.3
Refer to GI for further evaluation
(Remember: 4 = 1+3)

## Slide 17
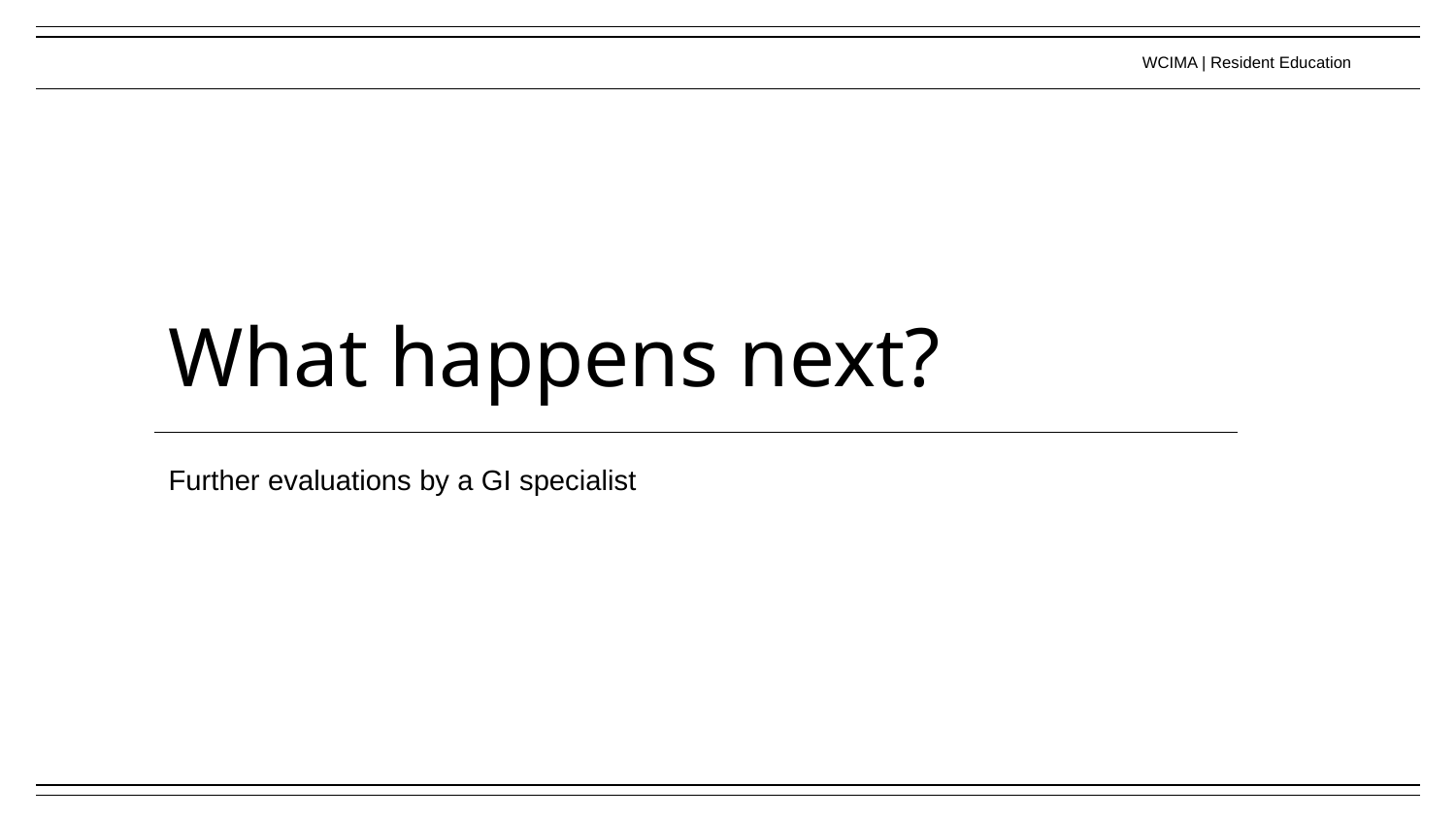

WCIMA | Resident Education
# What happens next?
Further evaluations by a GI specialist

## Slide 18
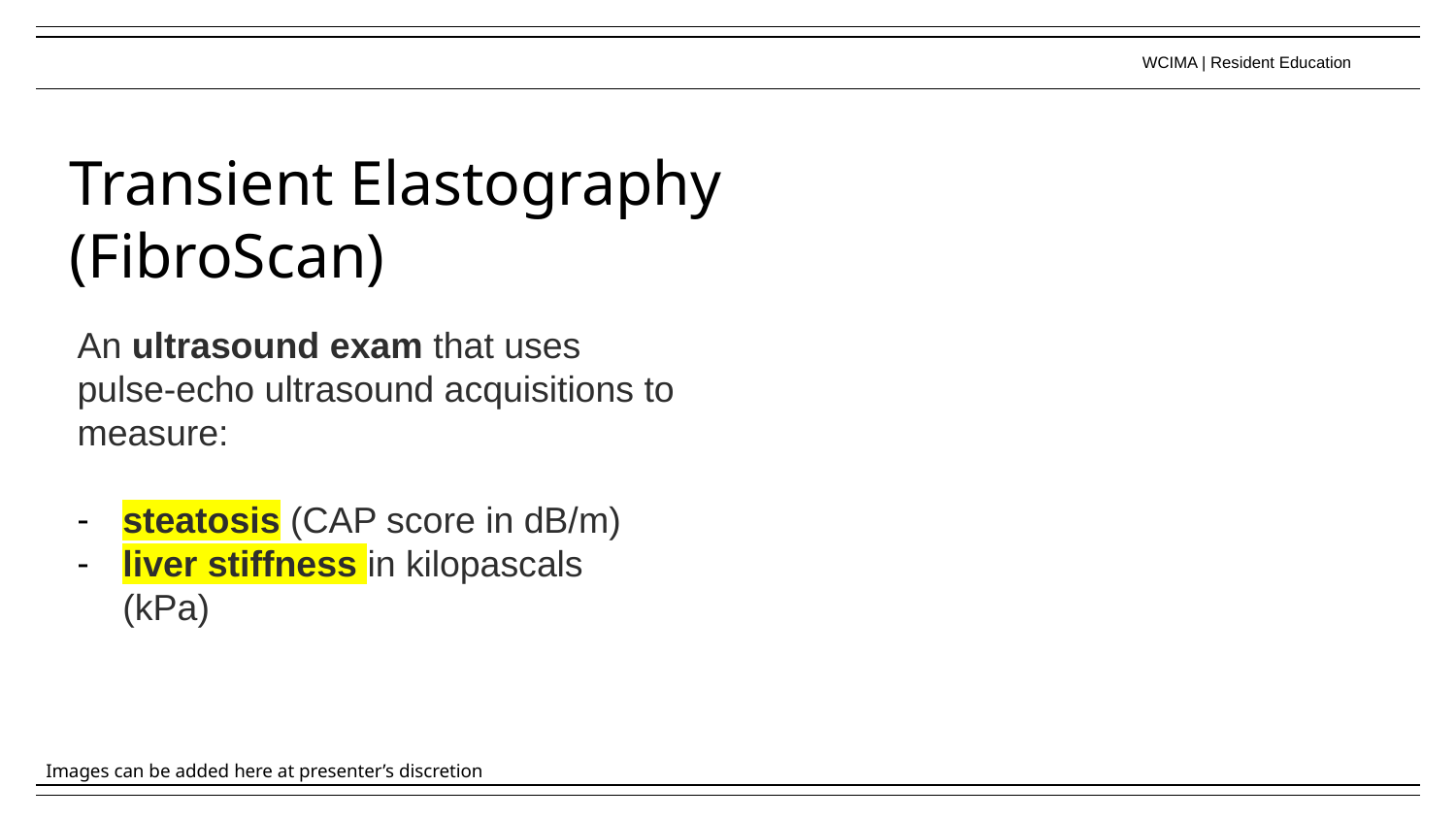

WCIMA | Resident Education
# Transient Elastography (FibroScan)
An ultrasound exam that uses pulse-echo ultrasound acquisitions to measure:
steatosis (CAP score in dB/m)
liver stiffness in kilopascals (kPa)
Images can be added here at presenter’s discretion

## Slide 19
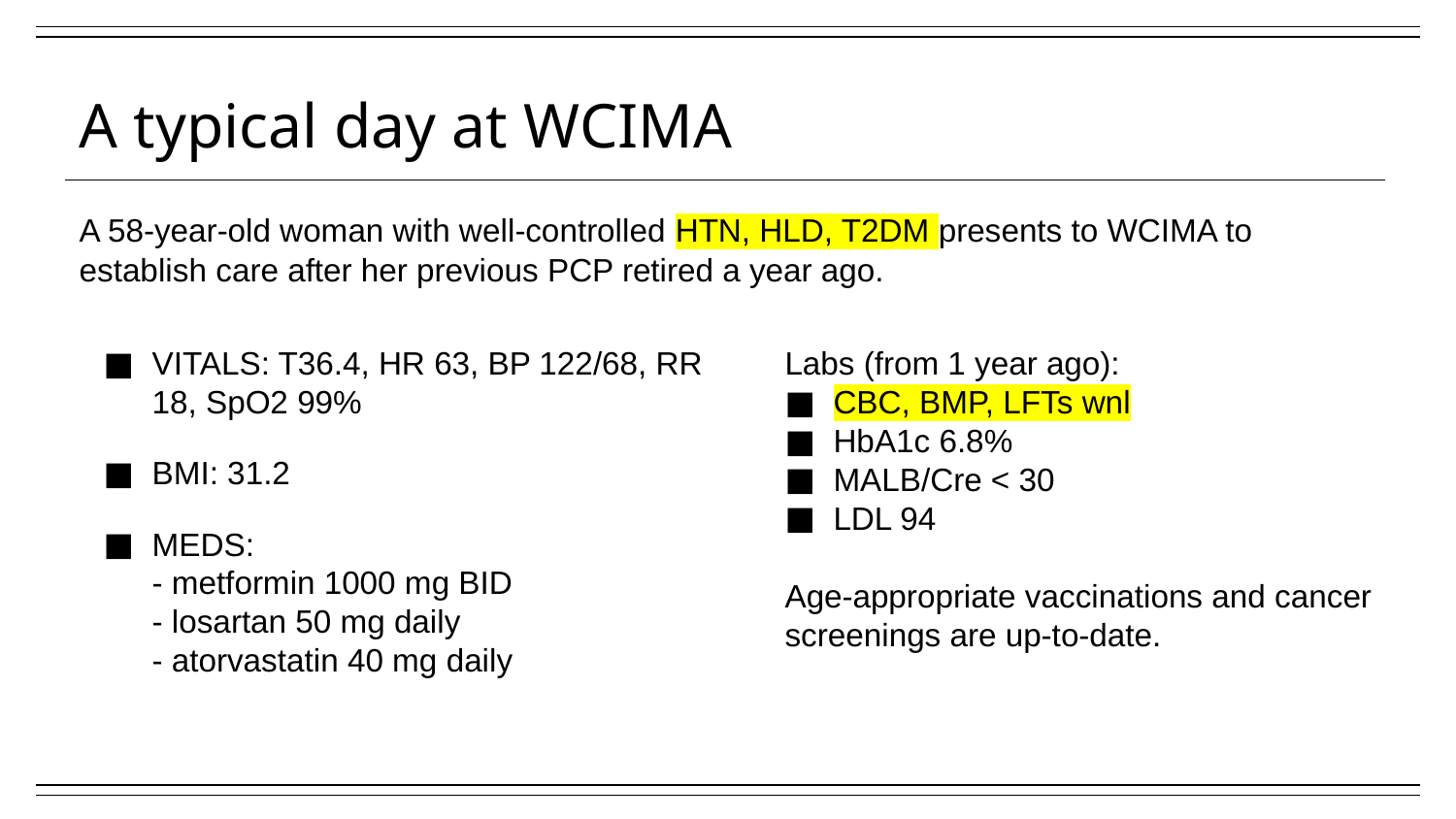

# A typical day at WCIMA
A 58-year-old woman with well-controlled HTN, HLD, T2DM presents to WCIMA to establish care after her previous PCP retired a year ago.
VITALS: T36.4, HR 63, BP 122/68, RR 18, SpO2 99%
BMI: 31.2
MEDS:
- metformin 1000 mg BID
- losartan 50 mg daily
- atorvastatin 40 mg daily
Labs (from 1 year ago):
CBC, BMP, LFTs wnl
HbA1c 6.8%
MALB/Cre < 30
LDL 94
Age-appropriate vaccinations and cancer screenings are up-to-date.

## Slide 20
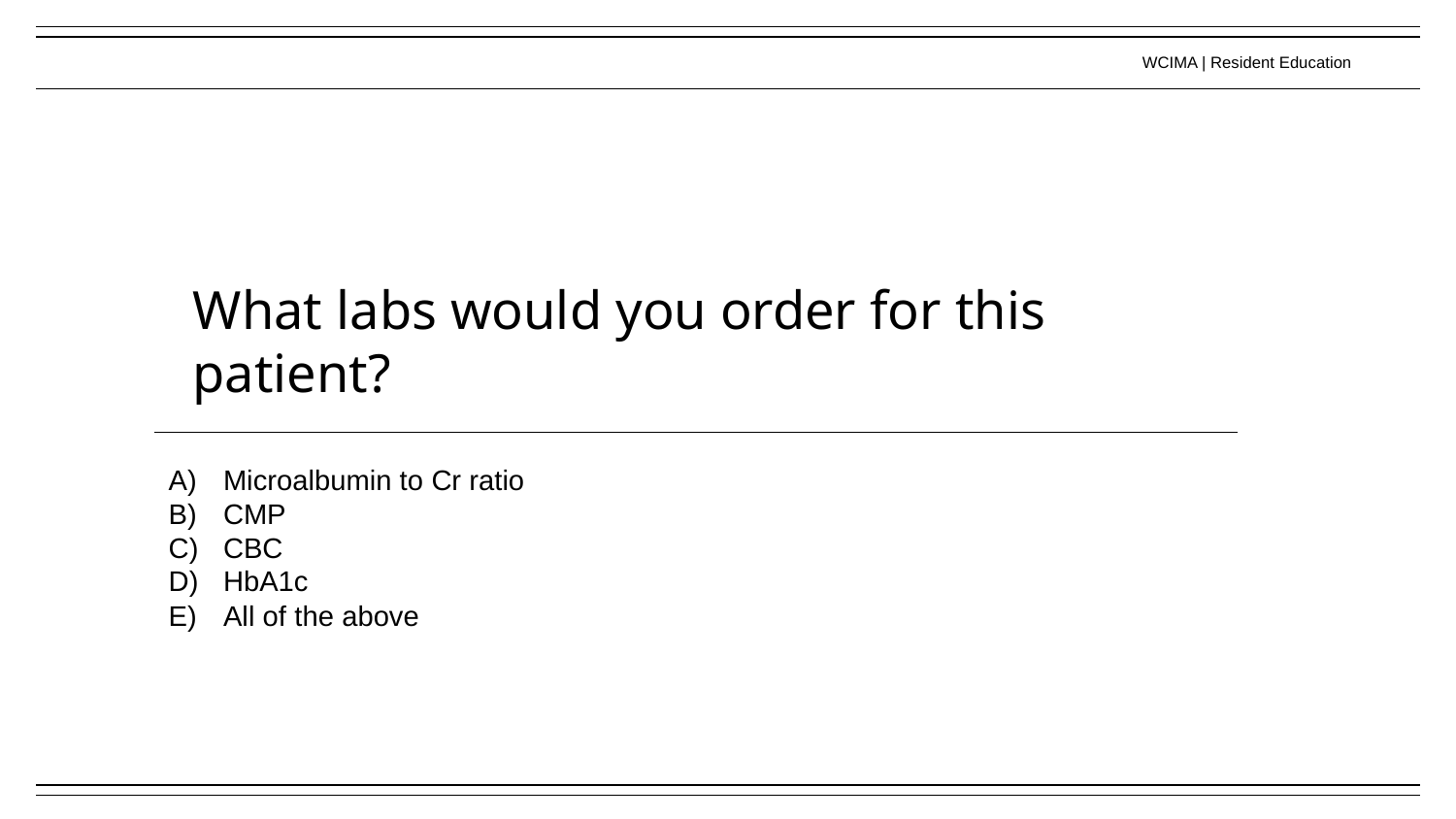

WCIMA | Resident Education
# What labs would you order for this patient?
Microalbumin to Cr ratio
CMP
CBC
HbA1c
All of the above

## Slide 21
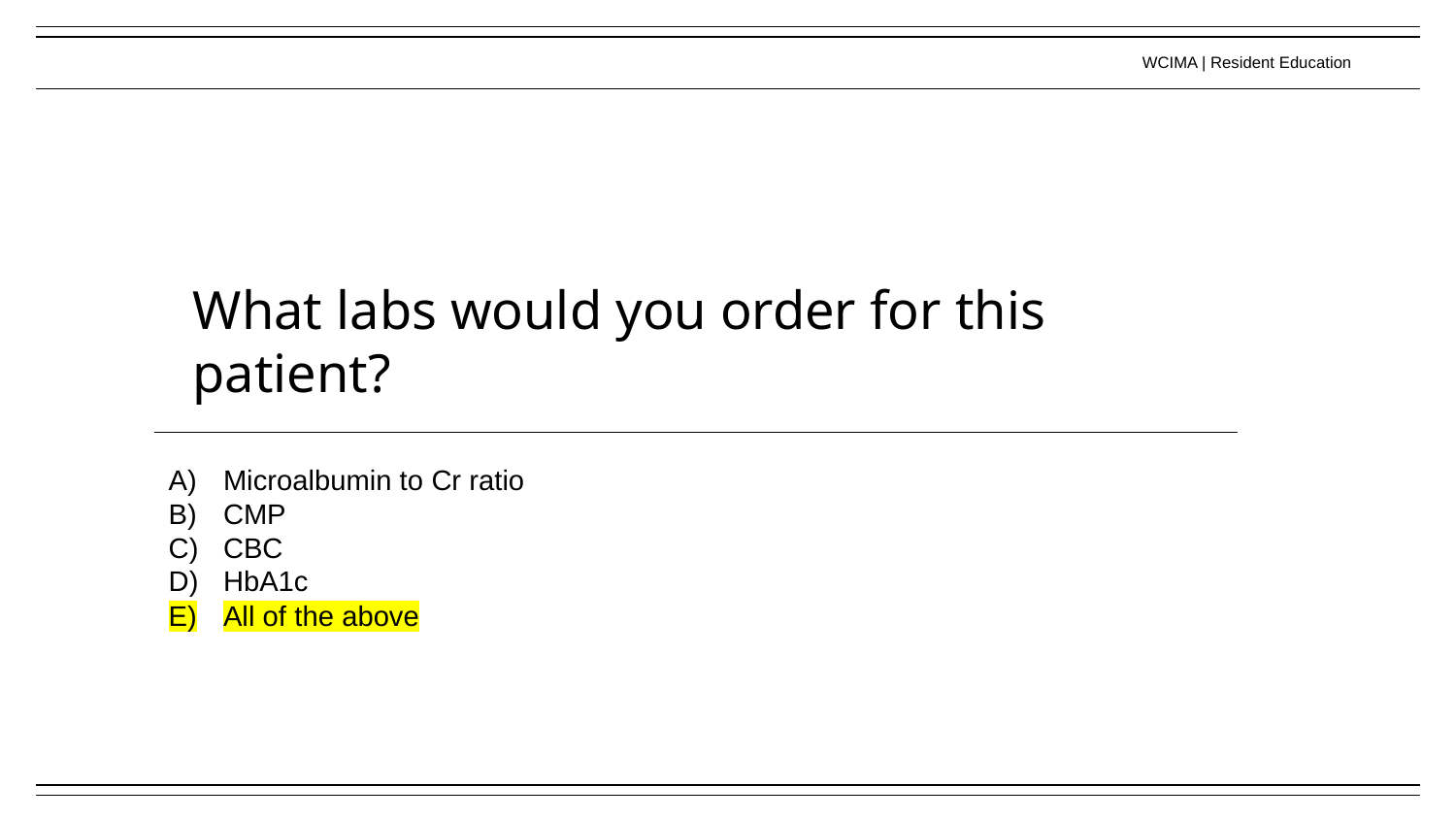

WCIMA | Resident Education
# What labs would you order for this patient?
Microalbumin to Cr ratio
CMP
CBC
HbA1c
All of the above

## Slide 22
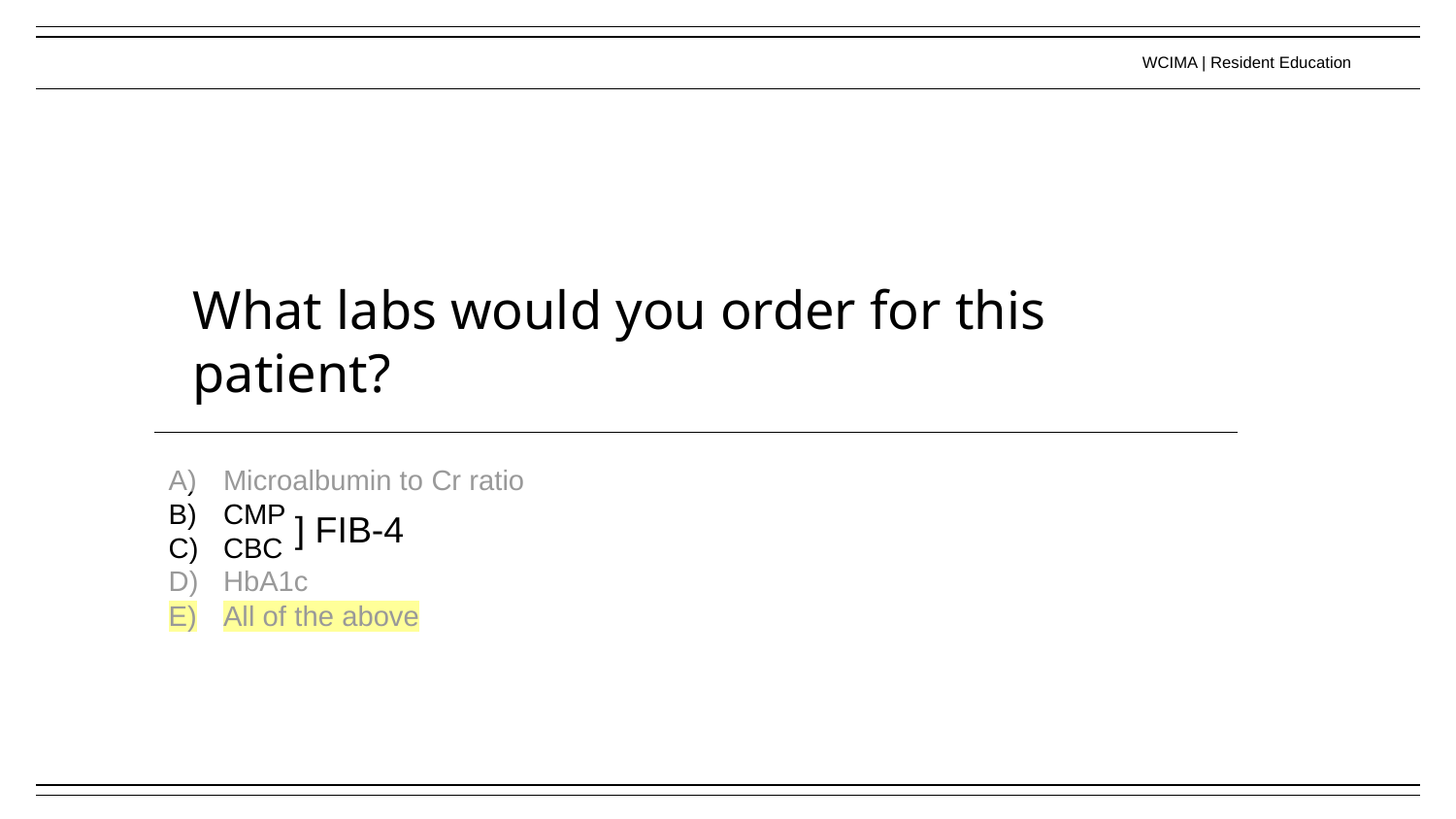

WCIMA | Resident Education
# What labs would you order for this patient?
Microalbumin to Cr ratio
CMP
CBC
HbA1c
All of the above
] FIB-4

## Slide 23
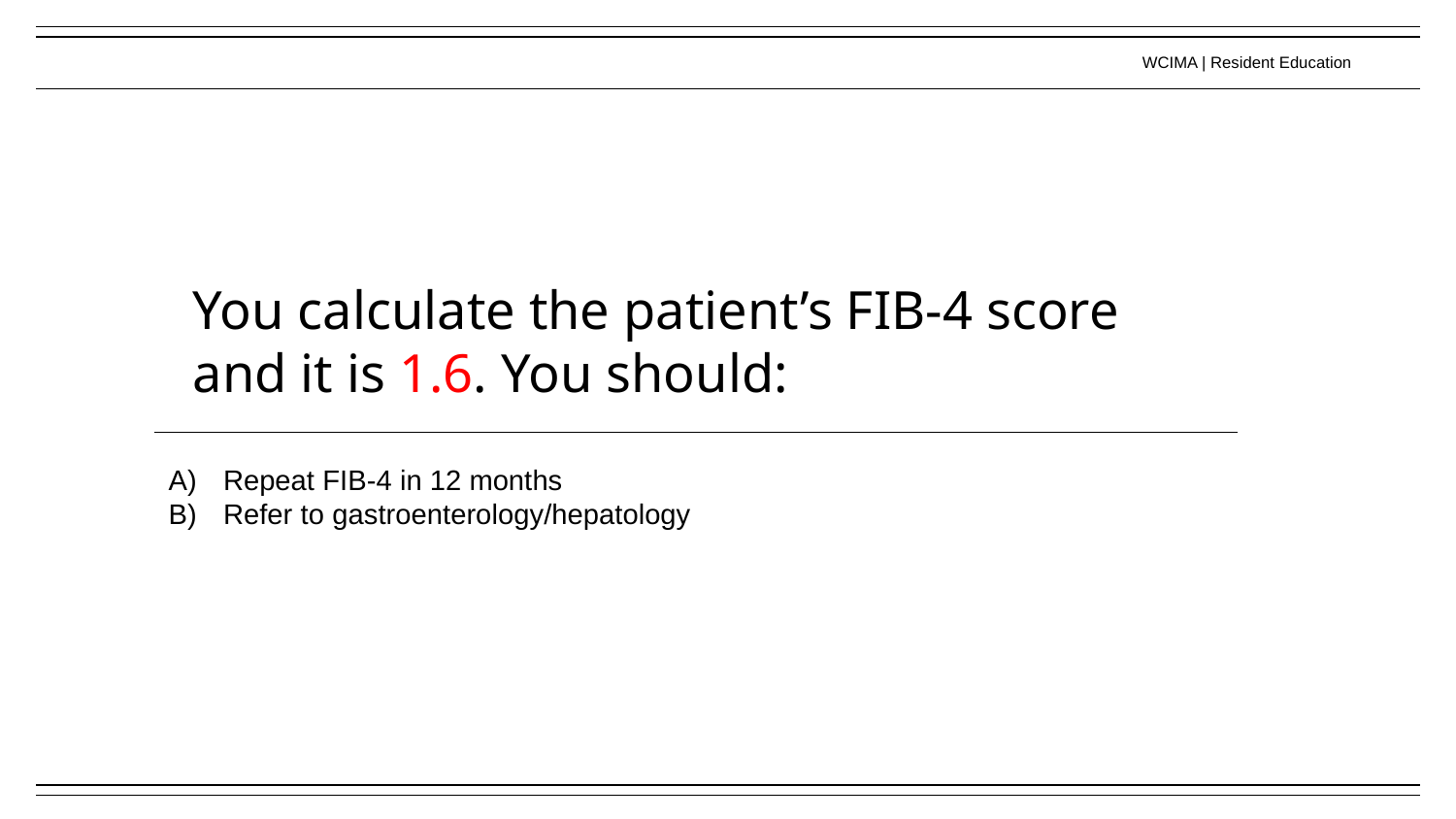

WCIMA | Resident Education
# You calculate the patient’s FIB-4 score and it is 1.6. You should:
Repeat FIB-4 in 12 months
Refer to gastroenterology/hepatology

## Slide 24
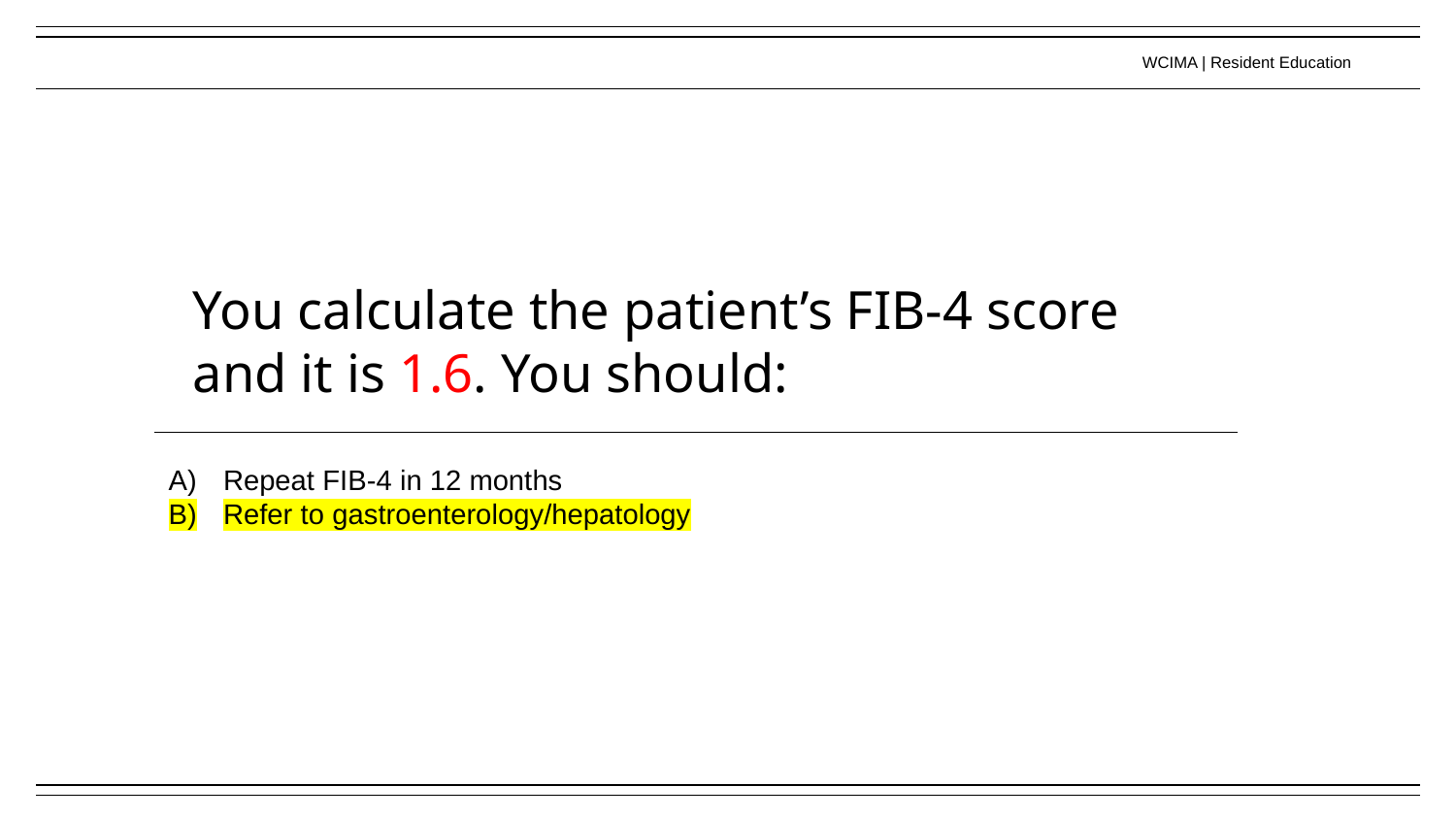

WCIMA | Resident Education
# You calculate the patient’s FIB-4 score and it is 1.6. You should:
Repeat FIB-4 in 12 months
Refer to gastroenterology/hepatology

## Slide 25
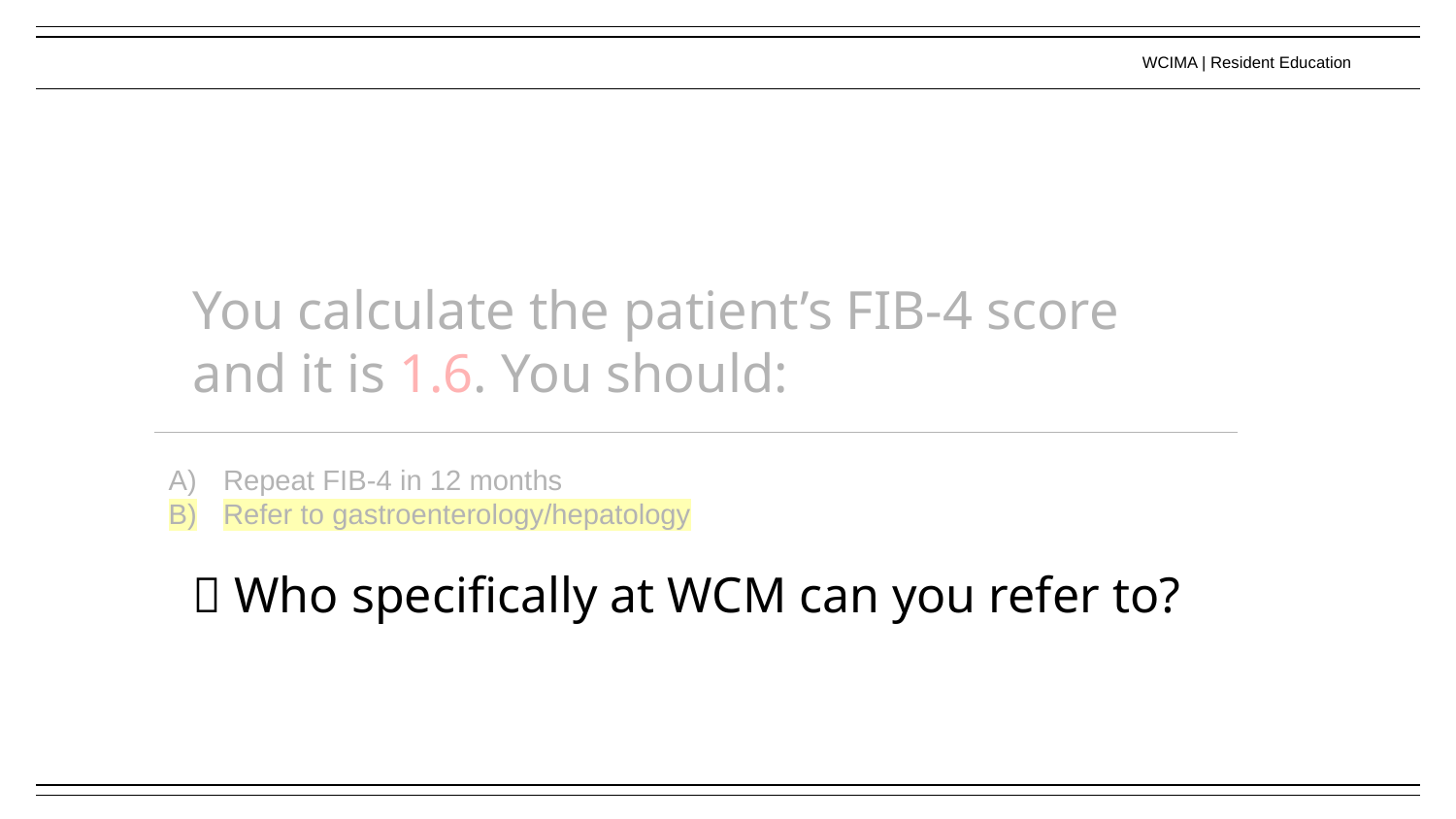

WCIMA | Resident Education
# You calculate the patient’s FIB-4 score and it is 1.6. You should:
Repeat FIB-4 in 12 months
Refer to gastroenterology/hepatology
  Who specifically at WCM can you refer to?

## Slide 26
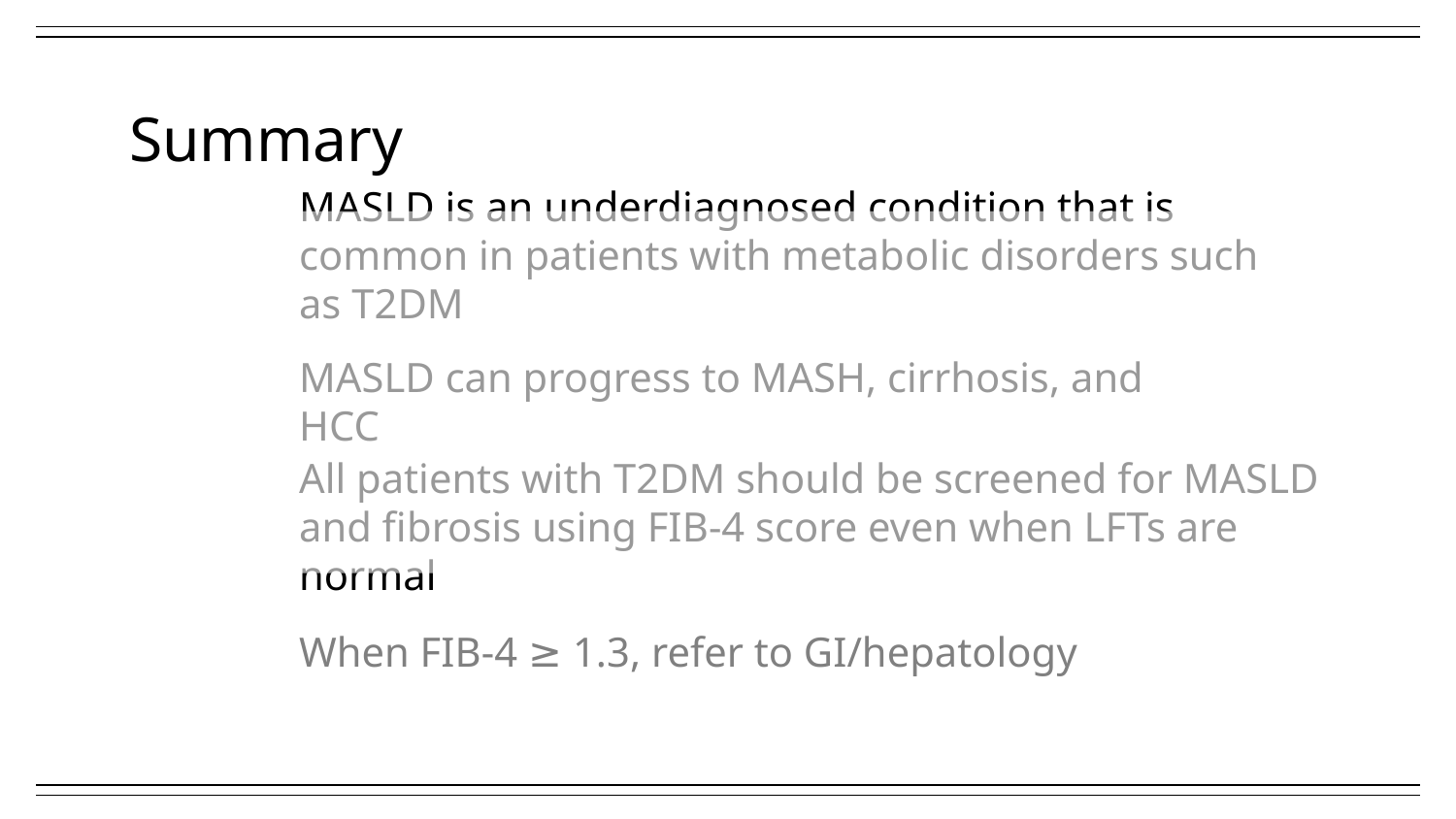

Summary
MASLD is an underdiagnosed condition that is common in patients with metabolic disorders such as T2DM
01
MASLD can progress to MASH, cirrhosis, and HCC
02
All patients with T2DM should be screened for MASLD and fibrosis using FIB-4 score even when LFTs are normal
03
When FIB-4 ≥ 1.3, refer to GI/hepatology
04

## Slide 27
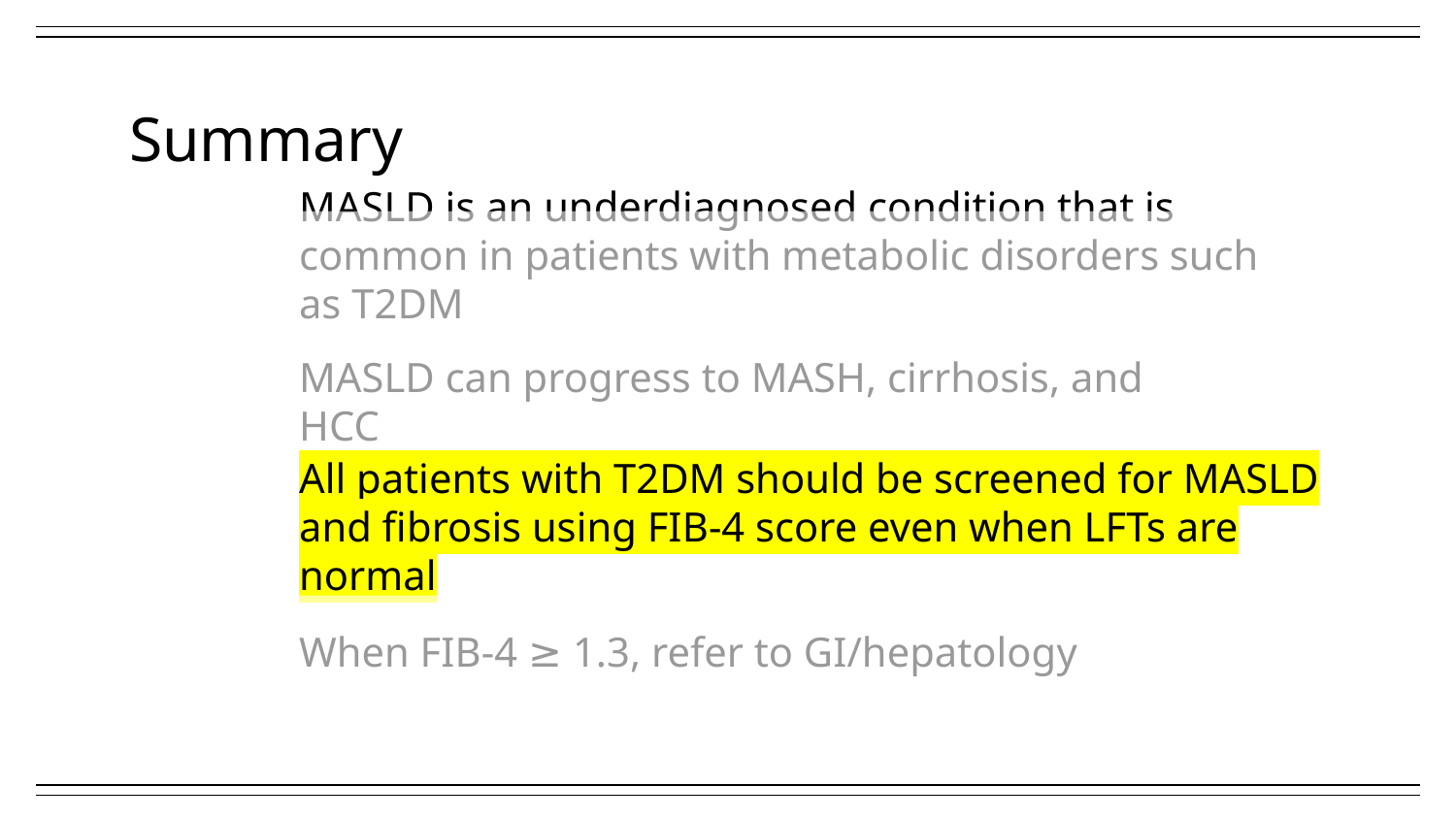

Summary
MASLD is an underdiagnosed condition that is common in patients with metabolic disorders such as T2DM
01
MASLD can progress to MASH, cirrhosis, and HCC
02
All patients with T2DM should be screened for MASLD and fibrosis using FIB-4 score even when LFTs are normal
03
When FIB-4 ≥ 1.3, refer to GI/hepatology
04

## Slide 28
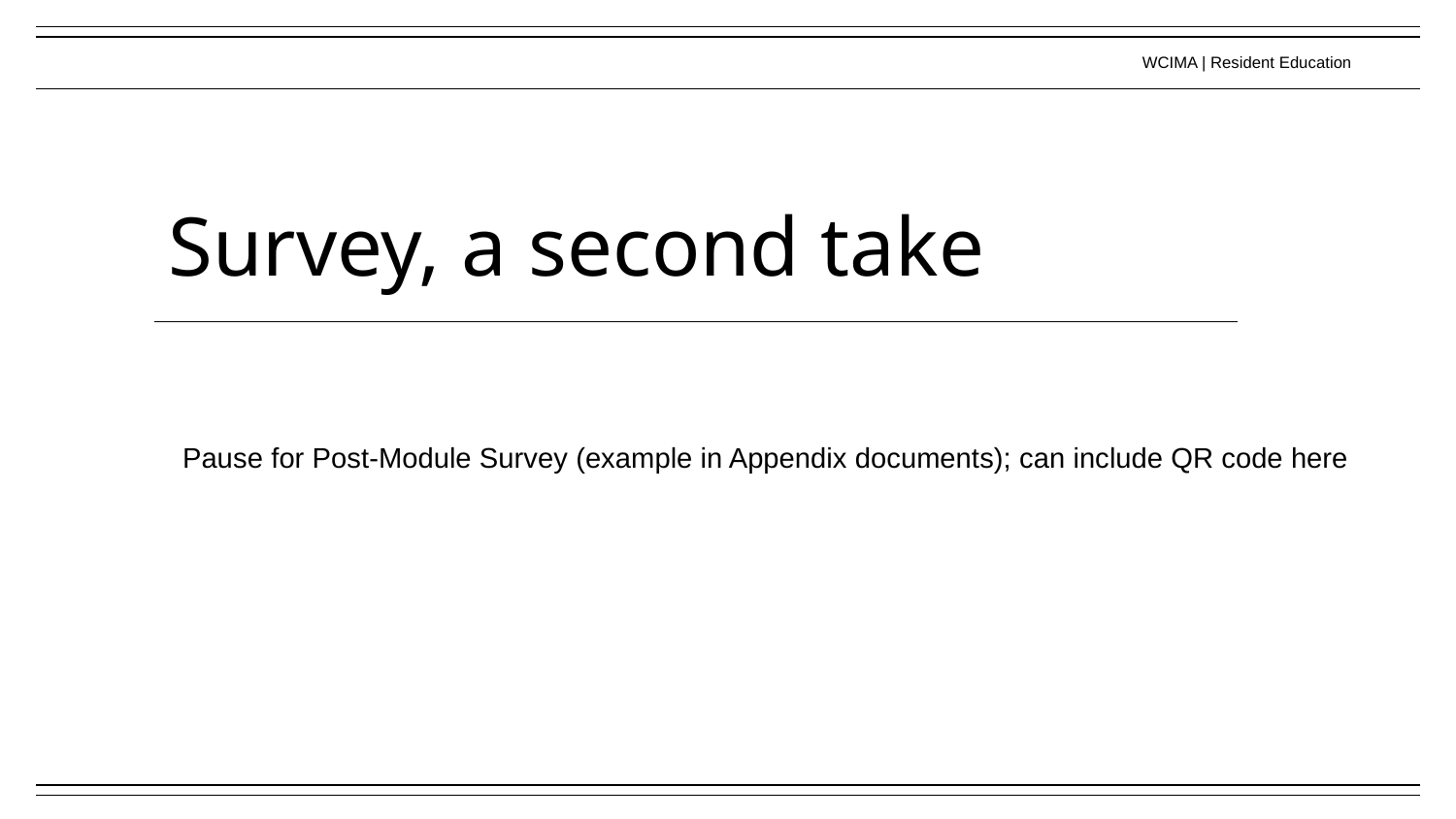

WCIMA | Resident Education
# Survey, a second take
Pause for Post-Module Survey (example in Appendix documents); can include QR code here

## Slide 29
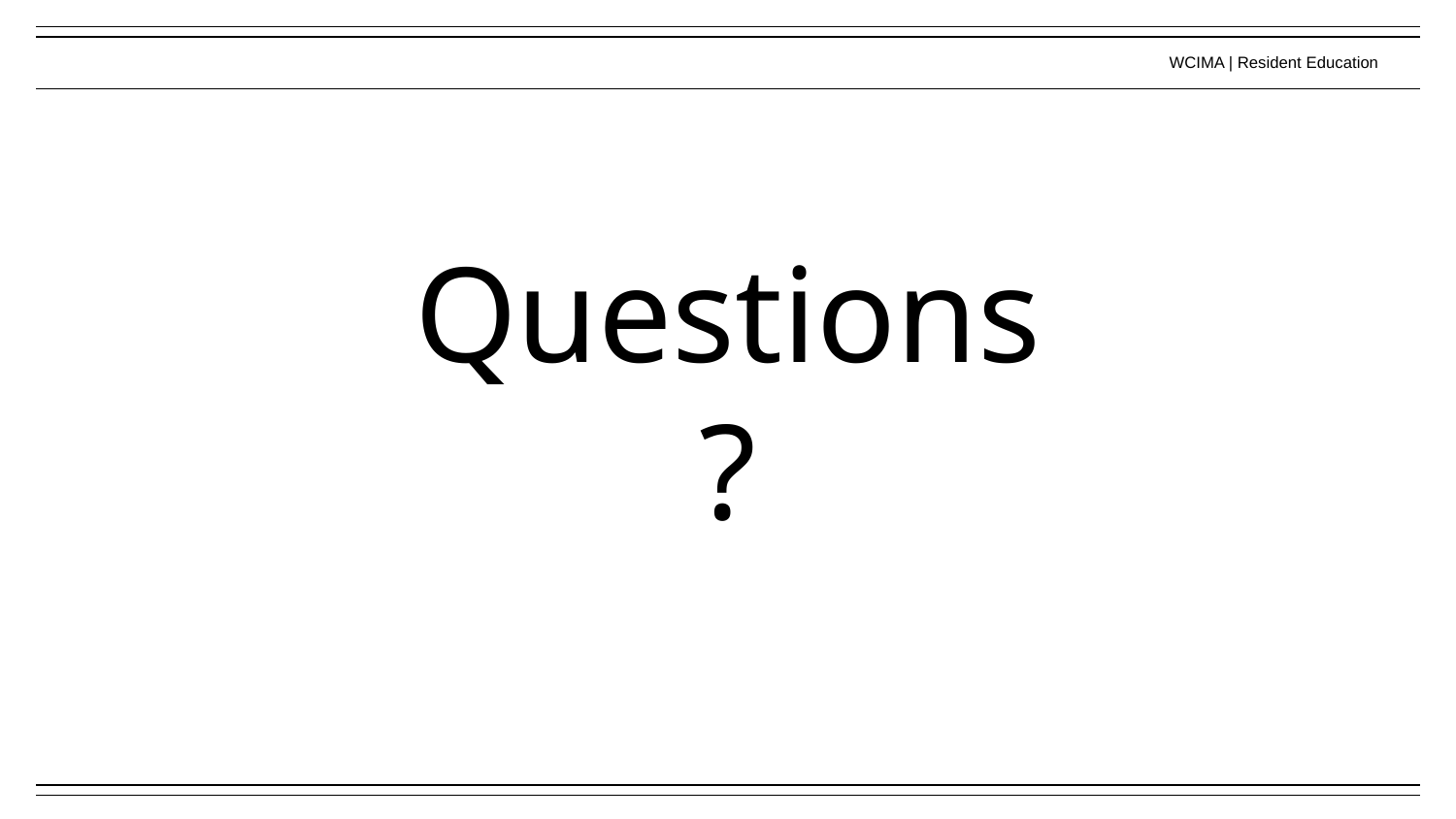

WCIMA | Resident Education
# Questions?

## Slide 30
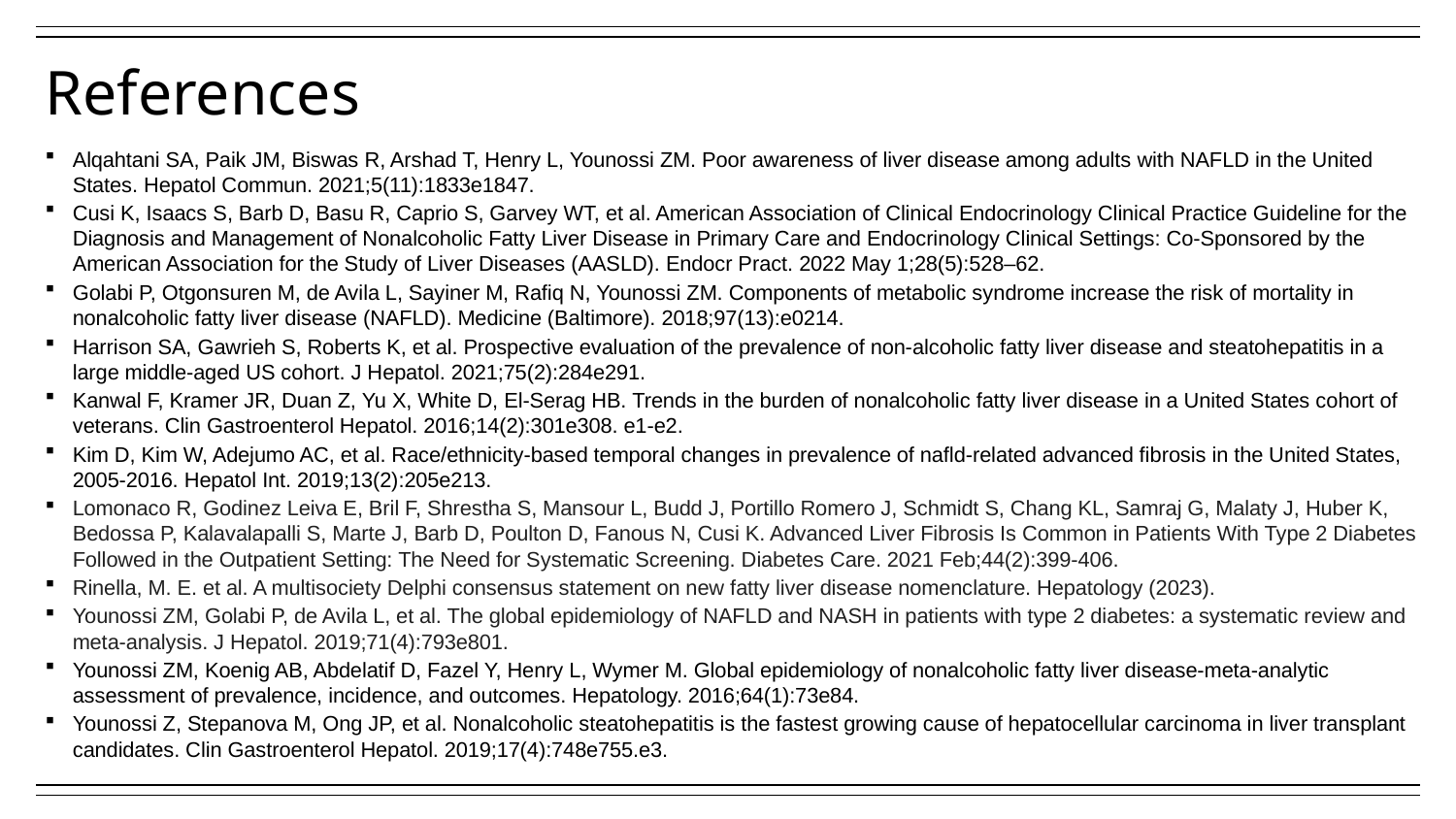

# References
Alqahtani SA, Paik JM, Biswas R, Arshad T, Henry L, Younossi ZM. Poor awareness of liver disease among adults with NAFLD in the United States. Hepatol Commun. 2021;5(11):1833e1847.
Cusi K, Isaacs S, Barb D, Basu R, Caprio S, Garvey WT, et al. American Association of Clinical Endocrinology Clinical Practice Guideline for the Diagnosis and Management of Nonalcoholic Fatty Liver Disease in Primary Care and Endocrinology Clinical Settings: Co-Sponsored by the American Association for the Study of Liver Diseases (AASLD). Endocr Pract. 2022 May 1;28(5):528–62.
Golabi P, Otgonsuren M, de Avila L, Sayiner M, Raﬁq N, Younossi ZM. Components of metabolic syndrome increase the risk of mortality in nonalcoholic fatty liver disease (NAFLD). Medicine (Baltimore). 2018;97(13):e0214.
Harrison SA, Gawrieh S, Roberts K, et al. Prospective evaluation of the prevalence of non-alcoholic fatty liver disease and steatohepatitis in a large middle-aged US cohort. J Hepatol. 2021;75(2):284e291.
Kanwal F, Kramer JR, Duan Z, Yu X, White D, El-Serag HB. Trends in the burden of nonalcoholic fatty liver disease in a United States cohort of veterans. Clin Gastroenterol Hepatol. 2016;14(2):301e308. e1-e2.
Kim D, Kim W, Adejumo AC, et al. Race/ethnicity-based temporal changes in prevalence of naﬂd-related advanced ﬁbrosis in the United States, 2005-2016. Hepatol Int. 2019;13(2):205e213.
Lomonaco R, Godinez Leiva E, Bril F, Shrestha S, Mansour L, Budd J, Portillo Romero J, Schmidt S, Chang KL, Samraj G, Malaty J, Huber K, Bedossa P, Kalavalapalli S, Marte J, Barb D, Poulton D, Fanous N, Cusi K. Advanced Liver Fibrosis Is Common in Patients With Type 2 Diabetes Followed in the Outpatient Setting: The Need for Systematic Screening. Diabetes Care. 2021 Feb;44(2):399-406.
Rinella, M. E. et al. A multisociety Delphi consensus statement on new fatty liver disease nomenclature. Hepatology (2023).
Younossi ZM, Golabi P, de Avila L, et al. The global epidemiology of NAFLD and NASH in patients with type 2 diabetes: a systematic review and meta-analysis. J Hepatol. 2019;71(4):793e801.
Younossi ZM, Koenig AB, Abdelatif D, Fazel Y, Henry L, Wymer M. Global epidemiology of nonalcoholic fatty liver disease-meta-analytic assessment of prevalence, incidence, and outcomes. Hepatology. 2016;64(1):73e84.
Younossi Z, Stepanova M, Ong JP, et al. Nonalcoholic steatohepatitis is the fastest growing cause of hepatocellular carcinoma in liver transplant candidates. Clin Gastroenterol Hepatol. 2019;17(4):748e755.e3.
