# Supplementary material for: From Guidelines to Practice: Case-Based Teaching Module to Improve MASLD Screening in Diabetes Care for Internal Medicine Residents
Source: MedEdPORTAL. 2026 Mar 24;22:11586. doi: 10.15766/mep_2374-8265.11586 (PMC13008821; doi:10.15766/mep_2374-8265.11586)
Supplement: Supplementary file 1 — MASLD Screening Education Module.pptxMASLD Survey.docx [file mep_2374-8265.11586-s001.zip › _Educational Summary Report_11586.pdf]

MedEdPORTAL
THE AAMC JOURNAL OF TEACHING AND LEARNING RESOURCES

Original Publication

Open Access

From Guidelines to Practice: Case-Based Teaching Module to Improve MASLD Screening in Diabetes Care for Internal Medicine Residents

Bethina Liu, MD†, Chimsom Orakwue, MD†, Kara Ryan, MD, Melina I. Manolas, MD, MPH, Sonal Kumar, MD, MPH*

*Corresponding author: sok9028@med.cornell.edu

†Coprimary authors

Abstract

Introduction: Metabolic dysfunction–associated steatotic liver disease (MASLD) is a prevalent, yet underrecognized condition with serious, but preventable, complications. The American Association for the Study of Liver Diseases, American Association of Clinical Endocrinology (2022), and American Diabetes Association (2025) recommend MASLD screening for patients with type 2 diabetes, including using the Fibrosis-4 (FIB-4) index for noninvasive risk stratification of those needing further evaluation. Primary care physicians are key to early diagnosis but may be unfamiliar with updated guidelines. **Methods:** A 30-minute case-based educational session on MASLD was delivered to 54 internal medicine residents in small groups during 2023–2024. Sessions integrated retrieval-based learning, brief didactics, and facilitated discussion. Participants completed 3 surveys (presession, immediately postsession, and year's end) assessing MASLD knowledge and perceived clinical impact. Scores (correct answer = 1 point, 12 total) overall and by category (Epidemiology, Practices) were analyzed by ANOVA and paired *t* tests. **Results:** Among 47 survey 1 respondents, 41 survey 2 respondents, and 9 survey 3 respondents, respective mean scores were 6.57, 9.20, and 8.00 overall ($p < .001$); 1.91, 3.88, and 2.56 for Epidemiology ($p < .001$); and 4.66, 5.32, and 5.44 for Practices ($p = .006$). Mean scores increased on survey 2 (overall +2.59; Epidemiology +1.95; Practices +0.63; each $p < .001$). Survey 3 mean scores increased for Practices (+1.22, $p = .01$), with 71% reporting screening changes and 76% reporting referral practice changes. **Discussion:** Targeted interventions improved knowledge of guideline-based MASLD screening, FIB-4 interpretation, and indications for hepatology referral.

Keywords
MASLD, Type 2 Diabetes, FIB-4, Cirrhosis, Metabolic Syndrome, Nutrition, Gastroenterology, Internal Medicine, Case-Based Learning

Educational Objectives

By the end of this activity, learners will be able to:

1. Identify metabolic dysfunction–associated steatotic liver disease (MASLD) as an underdiagnosed condition with significant public health impact, particularly among patients with diabetes.
2. Describe the major complications of MASLD, including metabolic dysfunction–associated steatohepatitis, cirrhosis, and hepatocellular carcinoma, using evidence-based epidemiologic data.
3. Apply guideline-based approaches to screening for MASLD and advanced fibrosis in the primary care setting.
4. Calculate and interpret the Fibrosis-4 score to determine whether a patient with type 2 diabetes meets criteria for further MASLD workup or specialist referral.

Citation:
Liu B, Orakwue C, Ryan K, Manolas MI, Kumar S. From guidelines to practice: case-based teaching module to improve MASLD screening in diabetes care for internal medicine residents. *MedEdPORTAL*. 2026;22:11586. https://doi.org/10.15766/mep_2374-8265.11586

Introduction

Metabolic dysfunction–associated steatotic liver disease (MASLD) is a growing public health concern, with a global prevalence of 25%, and a prevalence of up to 70% among individuals with type 2 diabetes (T2D).[1,2] MASLD can progress to metabolic dysfunction–associated steatohepatitis (MASH) and cirrhosis, and may predispose individuals to hepatocellular carcinoma. However, fewer than 5% of patients with MASLD report awareness of their diagnosis, and screening by primary care physicians remains low, even for patients with metabolic risk factors (66% for diabetes, 33% for hypertension, 38% for cardiovascular disease).[3,4] In 2022, the American Association for

Copyright © 2026 Liu et al. This is an open-access publication distributed under the terms of the Creative Commons Attribution license.

the Study of Liver Diseases (AASLD) and American Association of Clinical Endocrinology (AACE) issued MASLD screening guidelines, which recommend using the Fibrosis-4 (FIB-4) index to screen for advanced fibrosis in all patients with T2D, regardless of liver enzyme levels.[5,6] The American Diabetes Association 2025 guidelines reinforced these recommendations. Calculated using readily available clinical data (age, aspartate aminotransferase levels, alanine aminotransferase levels, and platelet count), the FIB-4 index has a 90% negative predictive value for risk of advanced fibrosis for those with a score of <1.3, and an 80% positive predictive value for risk of advanced fibrosis for those with a score of >2.67.[7,8] Those with an FIB-4 index score indicating indeterminate risk (scores 1.3–2.67) or high risk (scores >2.67) of advanced fibrosis should receive further testing or evaluation by a specialist.[5]

Primary care physicians are critical for early identification and prevention of disease progression, but competing priorities and limited exposure to hepatology or endocrinology guidelines may hinder consistent screening and referral.[9] Education efforts are urgently needed to improve guideline-based practice, especially as the therapeutic armamentarium expands, including the recent US Food and Drug Administration approval of resmetirom and semaglutide as treatments for noncirrhotic MASH with moderate-to-advanced fibrosis. To address this need, we developed an interactive education module for internal medicine (IM) residents at New York-Presbyterian/Weill Cornell Medicine (NYP/WCM), aiming to improve provider awareness of MASLD guidelines for patients with T2D and impact clinical practice.

A *MedEdPORTAL* search for the terms *cirrhosis* and *MASLD* (formerly, nonalcoholic fatty liver disease) yielded only 1 educational intervention, which focused on teaching ascites diagnosis and management at the medical student level. In addition, there currently are no curricula published on *MedEdPORTAL* with the new nomenclature that addresses the recent redefinition and evolving guidelines in the context of primary care for high-risk populations such as those with T2D. Our novel curriculum addressed this gap by integrating current AACE and AASLD screening recommendations for MASLD into a case-based, small-group format specifically designed for IM residents in the ambulatory setting.

Methods

The curriculum was developed in response to new MASLD screening guidelines to increase provider awareness in the primary care setting. It was developed by one of the authors (Bethina Liu) under the mentorship of another author (Sonal Kumar), in partnership with Weill Cornell Internal Medicine Associates leadership (Kara Ryan), and with feedback from individuals in the medical education space. The design of this module was grounded in several educational theories, including the following: (1) retrieval-based learning,[10] involving knowledge checks and multiple-choice questions embedded throughout the session, to promote active recall, which enhances knowledge retention; (2) case-based learning frameworks,[11] which anchors concepts within a real clinical scenario to support clinical reasoning and application; (3) situated learning theory,[12] proposing that the module be delivered immediately before clinic encounters to allow learners to apply new knowledge to real patients, thereby enhancing knowledge transfer and retention; and (4) constructivist pedagogy,[13] in which small-group discussions and think-pair-share activities promote learner engagement through peer dialogue and co-construction of understanding. To our knowledge, this is one of the first resident-level educational interventions to address MASLD using updated nomenclature, contemporary guidelines, and a practice-integrated instructional design.

All IM residents in training at NYP/WCM during the 2023–2024 academic year ($N = 139$) were eligible to participate in the educational intervention. PGY-1 and PGY-3 residents received the intervention during their outpatient ambulatory rotation; PGY-2 residents, who follow a continuity clinic schedule, participated during didactic time on clinic days. Institutional review board exemption was obtained prior to implementing the session (Assessing the Effectiveness of Non-Alcoholic Fatty Liver Disease Screening Education Among Residents in the Primary Care Setting; exemption received June 2023).

We developed a 30-minute interactive, case-based education session (Appendix A) focused on 4 educational objectives: (1) to recognize MASLD as an underdiagnosed condition with significant public health implications; (2) to describe the complications of MASLD; (3) to assess when and how to screen for MASLD and clinically significant fibrosis in the primary care setting; and (4) to identify hepatology referral criteria using the FIB-4 index. Sessions were conducted primarily in small groups of 2–4 residents to promote active discussion and peer engagement. Two larger sessions, each consisting of ~15 residents, were conducted using think-pair-share techniques to encourage active participation. Due to the preestablished structural differences in the residency schedules, PGY-1 and PGY-3 residents follow an X + Y (X being inpatient weeks and Y being outpatient weeks) ambulatory block curriculum, which allowed entire cohorts to receive the session only during their scheduled Y-weeks. In contrast, PGY-2 residents do not have protected ambulatory blocks but follow a weekly continuity clinic model

Copyright © 2026 Liu et al. This is an open-access publication distributed under the terms of the Creative Commons Attribution license.

with 30-minute didactic time on clinic days. On any given day, between 2 and 4 PGY-2 residents participated in the continuity clinic. Therefore, our session was delivered to PGY-1 and PGY-3 residents in larger groups ($n = 15$) during their structured outpatient morning conference, and to PGY-2 residents in small groups ($n = 2$–4) during continuity clinic didactics. Overall, more PGY-2 residents than PGY-1 and PGY-3 residents received the session.

The chief resident of the ambulatory block facilitated each session, beginning with a clinical case on health care maintenance in a patient with T2D, to stimulate discussion and highlight current practice patterns. This was followed by a structured PowerPoint presentation covering: (1) diagnostic criteria, pathogenesis, and complications of MASLD; (2) epidemiology and risk factors associated with MASLD; and (3) a focused review of AACE and AASLD guidelines, emphasizing screening recommendations for patients with T2D. Embedded multiple-choice questions were used throughout the presentation to reinforce key concepts. The session concluded with a return to the original case, allowing participants to apply newly acquired knowledge. The sessions were deliberately scheduled immediately prior to resident clinic, enabling prompt application of the material to real-world patient care.

Before each session, participants completed a baseline survey (survey 1; Appendix B) containing 12 multiple-choice questions to assess MASLD epidemiology knowledge (questions 1–5) and awareness of screening recommendations and current practice patterns (questions 6–12). Questions were adapted from a 52-question survey used in a study by Younossi et al in 2022 on global physician knowledge about MASLD to focus on US-based epidemiology and screening practices.[4] Two postsession surveys were administered, 1 immediately following the educational session (survey 2; Appendix B) and the other at the end of the academic year (survey 3; Appendix B). Both surveys contained the same 12 multiple-choice questions assessing MASLD epidemiology knowledge and awareness of screening recommendations and current practice patterns, with the addition of feedback questions on perceived improvement in medical knowledge (surveys 2 and 3) and self-reported impact on screening practice (survey 3 only). Voluntary and anonymous surveys were administered electronically using Qualtrics. Participants were prompted to self-generate a unique identifier when completing survey 1 and were asked to enter the same identifier on surveys 2 and 3 to allow anonymous submission and analysis across surveys. Subsequent surveys with nonmatching identifiers were excluded from analysis.

Multiple-choice survey questions required respondents to indicate an answer choice for each item, with scoring of each correct answer corresponding to 1 point (of 12 points total). Questions were categorized into Epidemiology (questions 1–5; 5 points total) and Practices (questions 6–12; 7 points total), with mean scores calculated both for overall and by category. ANOVA and paired *t* tests were used to detect statistically significant differences in mean scores across surveys.

This methodology was intentionally selected to align with established educational frameworks and to meet the practical constraints of the residency curriculum. A brief, case-based, small-group format was chosen because it supports active learning, promotes peer discussion, and allows learners to immediately apply new knowledge—a core principle of case-based learning frameworks and constructivist pedagogy. Embedding retrieval prompts and multiple-choice knowledge checks was informed by retrieval-based learning theory, which has been shown to strengthen knowledge retention. Additionally, scheduling sessions immediately before resident participation in clinic encounters drew on situated learning theory, allowing learners to integrate new MASLD screening recommendations into real-time patient care. Finally, the format was also selected for feasibility within the existing ambulatory block and continuity clinic schedules, making it possible to incorporate guideline-focused teaching without adding curricular burden.

Results

The module was delivered to 54 IM residents between November 2023 and June 2024, and surveys were requested from all in attendance. A total of 47 of the 54 participants completed survey 1, 45 (96%) completed survey 2, and 21 (45%) completed survey 3. Excluding responses without matching identifiers, 47 survey 1 responses, 41 survey 2 responses, and 9 survey 3 responses were included for analysis (Table 1). Median time between initial educational session and survey 3 completion was 159 days (interquartile range 121–161 days).

Overall, mean scores (of 12 points total) assessing MASLD epidemiology knowledge and awareness of screening recommendations and current practice patterns were 6.57, 9.20, and 8.00 on surveys 1, 2, and 3, respectively. In the category of Epidemiology, mean scores (of 5 points total) were 1.91, 3.88, and 2.56 on surveys 1, 2, and 3, respectively. In the Practices category, mean scores (of 7 points total) were 4.66, 5.32, and 5.44 on surveys 1, 2, and 3, respectively (Table 1). Significant differences in scores across surveys were detected by ANOVA,

Copyright © 2026 Liu et al. This is an open-access publication distributed under the terms of the Creative Commons Attribution license.

Table 1. Summary of Survey Results Among Internal Medicine Residents Participating in the Case-Based Teaching Module on MASLD Screening in Diabetes Care

		PGY			Epidemiology		Practices		Overall	
Survey[a]	*n*	1	2	3	*M (SD)*	*P*[b]	*M (SD)*	*P*[b]	*M (SD)*	*P*[b]
1	47	11	29	7	1.91 (1.19)	<.001	4.66 (1.03)	.006	6.57 (1.54)	<.001
2	41	9	25	7	3.88 (1.21)		5.32 (1.06)		9.20 (1.63)	
3	9	2	7	0	2.56 (1.13)		5.44 (0.88)		8.00 (1.22)	

Abbreviation: MASLD, metabolic dysfunction–associated steatotic liver disease.
[a]Survey 1 = presession; survey 2 = immediately postsession; survey 3 = at end of academic year.
[b]ANOVA was used to test for statistically significant differences among the mean scores across surveys.

for the overall scores ($p < .001$) as well as for the Epidemiology scores ($p < .001$) and Practices scores ($p = .006$).

Paired *t* test analyses comparing score changes across surveys (Table 2) showed increases in mean scores between survey 1 and survey 2, for overall scores (+2.59, 95% CI 1.99, 3.18; $p < .001$), for Epidemiology scores (+1.95, 95% CI 1.46, 2.44; $p < .001$), and for Practices scores (+0.63, 95% CI 0.35, 0.92; $p < .001$). From survey 2 to survey 3, Epidemiology mean scores decreased (−1.75, 95% CI −2.91, −0.59; $p = .009$), while mean scores overall and in the Practices category showed no significant change. Compared with mean scores on survey 1, survey 3 showed increased mean scores for Practices (+1.22, 95% CI 0.38, 2.06; $p = .01$) but with no significant change in mean scores for Epidemiology or overall (Table 2).

In response to feedback questions postsession, 100% and 76% of participants reported gaining new MASLD clinical knowledge on survey 2 and survey 3, respectively. On survey 3, 71% reported changes in screening practice and 76% reported changes in referral practice as a result of the education module (Table 3).

Discussion

Our educational intervention highlights the ongoing need for MASLD education at the primary care level, showing low baseline knowledge among IM residents. A brief case-based, interactive educational session, adapted from a validated survey and integrated into existing ambulatory rotation curricula, can improve short-term understanding of guideline-based screening and referral practices. High participation, interactive teaching methods, strong immediate follow-up, and improvements in practice-oriented knowledge support the feasibility and impact of this scalable intervention. These findings underscore the potential of graduate medical education programs to effectively incorporate specialty-focused guidelines into primary care training and drive timely, evidence-based care in evolving clinical landscapes. The significant immediate gains observed in both epidemiology and practice-oriented knowledge suggest that embedding retrieval-based questions and discussion within a clinical case is an effective approach for teaching evolving hepatology guidelines.

This intervention is limited by attrition in survey completion. The exclusion of surveys without matching identifiers from analysis—4 missing responses for survey 2 and 12 for survey 3—further reduced sample size. Because only 9 participants completed all 3 surveys with matching identifiers, findings related to sustained changes in knowledge and practice should be interpreted with caution. The limited number of matched pairs reduces the ability to draw firm conclusions about long-term retention and may introduce response bias. Participants with greater interest in MASLD may have been more likely to complete subsequent surveys. Timing variability in survey 3 may have impacted retention assessment, and self-reported behavior changes may not reflect actual clinical practice, although there was a mean score increase between survey 1 and survey 3 in the Practices category. As noted, the overrepresentation of PGY-2 residents—

Table 2. Comparison of Mean Scores Among Survey Respondents Before and After Participation in the Case-Based Teaching Module on MASLD Screening in Diabetes Care

	Epidemiology			Practices			Overall		
Survey Comparison[a]	Δ *M*	95% CI	*P*[b]	Δ *M*	95% CI	*P*[b]	Δ *M*	95% CI	*P*[b]
1 vs. 2	1.95	1.46, 2.44	<.001	0.63	0.35, 0.92	<.001	2.59	1.99, 3.18	<.001
2 vs. 3	−1.75	−2.91, −0.59	.009	0.38	−0.71, 1.46	.44	−1.38	−2.98, 0.23	.08
1 vs. 3	0.11	−1.19, 1.41	.85	1.22	0.38, 2.06	.01	1.33	−0.05, 2.72	.06

Abbreviation: MASLD, metabolic dysfunction–associated steatotic liver disease.
[a]Survey 1 = presession; survey 2 = immediately postsession; survey 3 = at end of academic year.
[b]Paired *t* test was used to detect statistically significant differences in mean scores.

Copyright © 2026 Liu et al. This is an open-access publication distributed under the terms of the Creative Commons Attribution license.

Table 3. Participant Responses to Feedback Questions After Participating in the Case-Based Teaching Module on MASLD Screening in Diabetes Care

Question	Survey 2[a] Agree *n* (%)	Survey 3[b] Agree *n* (%)
1. The MASLD module taught me new clinical knowledge.	45 (100)	16 (76)
2. I have changed my MASLD screening practices as a result of this module.	NA	15 (71)
3. I have changed my referral practices for patients with suspected MASLD as a result of this teaching module.	NA	16 (76)

Abbreviations: MASLD, metabolic dysfunction-associated steatotic liver disease; NA, not applicable.
[a]*N* = 45 (immediately postsession).
[b]*N* = 21 (at end of academic year).

stemming from the structure of the continuity clinic curriculum—may further affect the generalizability of the findings. Baseline knowledge and prior exposure to hepatology concepts likely vary by training level, which could also influence survey performance.

Despite these limitations, the intervention was feasible, positively received, and demonstrated immediate improvements in MASLD-related knowledge. Future work will examine objective clinical behaviors, such as documentation of FIB-4 scores and hepatology referrals, to determine whether knowledge gains translate into practice change.

Conclusion

A brief and targeted educational session meaningfully increased IM resident knowledge of MASLD screening and referral recommendations and influenced self-reported practice. Integrating guideline-based MASLD education into residency curriculum may enhance MASLD diagnosis and early intervention in primary care.

Appendices

A. MASLD Screening Education Module.pptx

B. MASLD Survey.docx

All appendices are peer reviewed as integral parts of the Original Publication.

Bethina Liu, MD: Fellow, Hematology and Oncology, Columbia University Irving Medical Center

Chimsom Orakwue, MD: Internal Medicine Third-Year Resident, New York-Presbyterian/Weill Cornell Medicine; ORCID: https://orcid.org/0009-0009-1274-0528

Kara Ryan, MD: Fellow, Addiction Medicine, Boston University Medical Center

Melina I. Manolas, MD, MPH: Assistant Professor, Department of Medicine, Division of Endocrinology, Diabetes & Metabolism, Weill Cornell Medicine

Sonal Kumar, MD, MPH: Assistant Professor of Medicine, Director of General Gastroenterology and Hepatology, Weill Cornell Medicine; ORCID: https://orcid.org/0000-0002-6873-6933

Disclosures

None to report.

Funding/Support

This work was supported through the Weill Cornell Medicine Primary Care Innovations Seed Grant 2023-2024.

Ethical Approval

The Weill Cornell Medicine Institution Institutional Review Board reviewed this project.

References

1. Younossi ZM, Koenig AB, Abdelatif D, Fazel Y, Henry L, Wymer M. Global epidemiology of nonalcoholic fatty liver disease-meta-analytic assessment of prevalence, incidence, and outcomes. *Hepatology*. 2016;64(1):73-84. https://doi.org/10.1002/hep.28431
2. Leung PB, Davis AM, Kumar S. Diagnosis and management of nonalcoholic fatty liver disease. *JAMA*. 2023;330(17):1687-1688. https://doi.org/10.1001/jama.2023.17935
3. Alqahtani SA, Paik JM, Biswas R, Arshad T, Henry L, Younossi ZM. Poor awareness of liver disease among adults with NAFLD in the United States. *Hepatol Commun*. 2021;5(11):1833-1847. https://doi.org/10.1002/hep4.1765
4. Younossi ZM, Ong JP, Takahashi H, et al; Global Nonalcoholic Steatohepatitis Council. A global survey of physicians knowledge about nonalcoholic fatty liver disease. *Clin Gastroenterol Hepatol*. 2022;20(6):e1456-e1468. https://doi.org/10.1016/j.cgh.2021.06.048
5. Cusi K, Isaacs S, Barb D, et al. American Association of Clinical Endocrinology clinical practice guideline for the diagnosis and management of nonalcoholic fatty liver disease in primary care and endocrinology clinical settings: co-sponsored by the American Association for the Study of Liver Diseases (AASLD). *Endocr Pract*. 2022;28(5):528-562. https://doi.org/10.1016/j.eprac.2022.03.010
6. Rinella ME, Neuschwander-Tetri BA, Siddiqui MS, et al. AASLD Practice Guidance on the clinical assessment and management of nonalcoholic fatty liver disease. *Hepatology*. 2023;77(5): 1797-1835. https://doi.org/10.1097/HEP.0000000000000323
7. Lee J, Vali Y, Boursier J, et al. Prognostic accuracy of FIB-4, NAFLD fibrosis score and APRI for NAFLD-related events: a

Copyright © 2026 Liu et al. This is an open-access publication distributed under the terms of the Creative Commons Attribution license.

systematic review. *Liver Int*. 2021;41(2):261-270. https://doi.org/10.1111/liv.14669

8. Kaltenbach M. Why are non-invasive risk scores such as FIB-4 used in clinical practice? American Association for the Study of Liver Diseases. 2022. Accessed February 6, 2026. https://www.aasld.org/liver-fellow-network/core-series/why-series/why-are-non-invasive-risk-scores-such-fib-4-used
9. Kokkorakis M, Boutari C, Hill MA, et al. Resmetirom, the first approved drug for the management of metabolic dysfunction-associated steatohepatitis: trials, opportunities, and challenges. *Metabolism*. 2024;154:155835. https://doi.org/10.1016/j.metabol.2024.155835
10. Karpicke JD, Lehman M, Aue WA. Chapter Seven—Retrieval-based learning: an episodic context account. *Psychol Learn Motiv*. 2014;61:237-284. https://doi.org/10.1016/B978-0-12-800283-4.00007-1
11. Tsekhmister Y. Effectiveness of case-based learning in medical and pharmacy education: a meta-analysis. *Electron J Gen Med*. 2023;20(5):em515. https://doi.org/10.29333/ejgm/13315
12. O'Brien BC, Battista A. Situated learning theory in health professions education research: a scoping review. *Adv Health Sci Educ Theory Pract*. 2020;25(2):483-509. https://doi.org/10.1007/s10459-019-09900-w
13. Thampinathan S. The application of the constructivism learning theory to physician assistant students in primary care. *Educ Health (Abingdon)*. 2022;35(1):26-30. https://doi.org/10.4103/efh.EfH_333_20

Received: August 10, 2025
Accepted: January 20, 2026
Published: March 24, 2026

Copyright © 2026 Liu et al. This is an open-access publication distributed under the terms of the Creative Commons Attribution license.
